# Resistance to androgen receptor signaling inhibition does not necessitate development of neuroendocrine prostate cancer

**DOI:** 10.1172/jci.insight.146827

**Published:** 2021-04-22

**Authors:** W. Nathaniel Brennen, Yezi Zhu, Ilsa M. Coleman, Susan L. Dalrymple, Lizamma Antony, Radhika A. Patel, Brian Hanratty, Roshan Chikarmane, Alan K. Meeker, S. Lilly Zheng, Jody E. Hooper, Jun Luo, Angelo M. De Marzo, Eva Corey, Jianfeng Xu, Srinivasan Yegnasubramanian, Michael C. Haffner, Peter S. Nelson, William G. Nelson, William B. Isaacs, John T. Isaacs

**Affiliations:** 1 Department of Oncology, Sidney Kimmel Comprehensive Cancer Center (SKCCC), Johns Hopkins University, Baltimore, Maryland, USA.; 2 Department of Urology, James Buchanan Brady Urological Institute, Johns Hopkins University School of Medicine, Baltimore, Maryland, USA.; 3 Division of Human Biology, Fred Hutchinson Cancer Research Center, Seattle, Washington, USA.; 4 Department of Pathology, SKCCC, Johns Hopkins University, Baltimore, Maryland, USA.; 5 Program for Personalized Cancer Care, North Shore University Health System, Evanston, Illinois, USA.; 6 Department of Urology and; 7 Department of Pathology, University of Washington, Seattle, Washington, USA.

**Keywords:** Oncology, Prostate cancer

## Abstract

Resistance to AR signaling inhibitors (ARSis) in a subset of metastatic castration-resistant prostate cancers (mCRPCs) occurs with the emergence of AR^–^ neuroendocrine prostate cancer (NEPC) coupled with mutations/deletions in *PTEN*, *TP53*, and *RB1* and the overexpression of DNMTs, EZH2, and/or SOX2. To resolve whether the lack of AR is the driving factor for the emergence of the NE phenotype, molecular, cell, and tumor biology analyses were performed on 23 xenografts derived from patients with PC, recapitulating the full spectrum of genetic alterations proposed to drive NE differentiation. Additionally, phenotypic response to CRISPR/Cas9-mediated AR KO in AR^+^ CRPC cells was evaluated. These analyses document that (a) ARSi-resistant NEPC developed without androgen deprivation treatment; (b) ARS in ARSi-resistant AR^+^/NE^+^ double-positive “amphicrine” mCRPCs did not suppress NE differentiation; (c) the lack of AR expression did not necessitate acquiring a NE phenotype, despite concomitant mutations/deletions in *PTEN* and *TP53*, and the loss of RB1 but occurred via emergence of an AR^–^/NE^–^ double-negative PC (DNPC); (d) despite DNPC cells having homogeneous genetic driver mutations, they were phenotypically heterogeneous, expressing basal lineage markers alone or in combination with luminal lineage markers; and (e) AR loss was associated with AR promoter hypermethylation in NEPCs but not in DNPCs.

## Introduction

**Figure 1:**
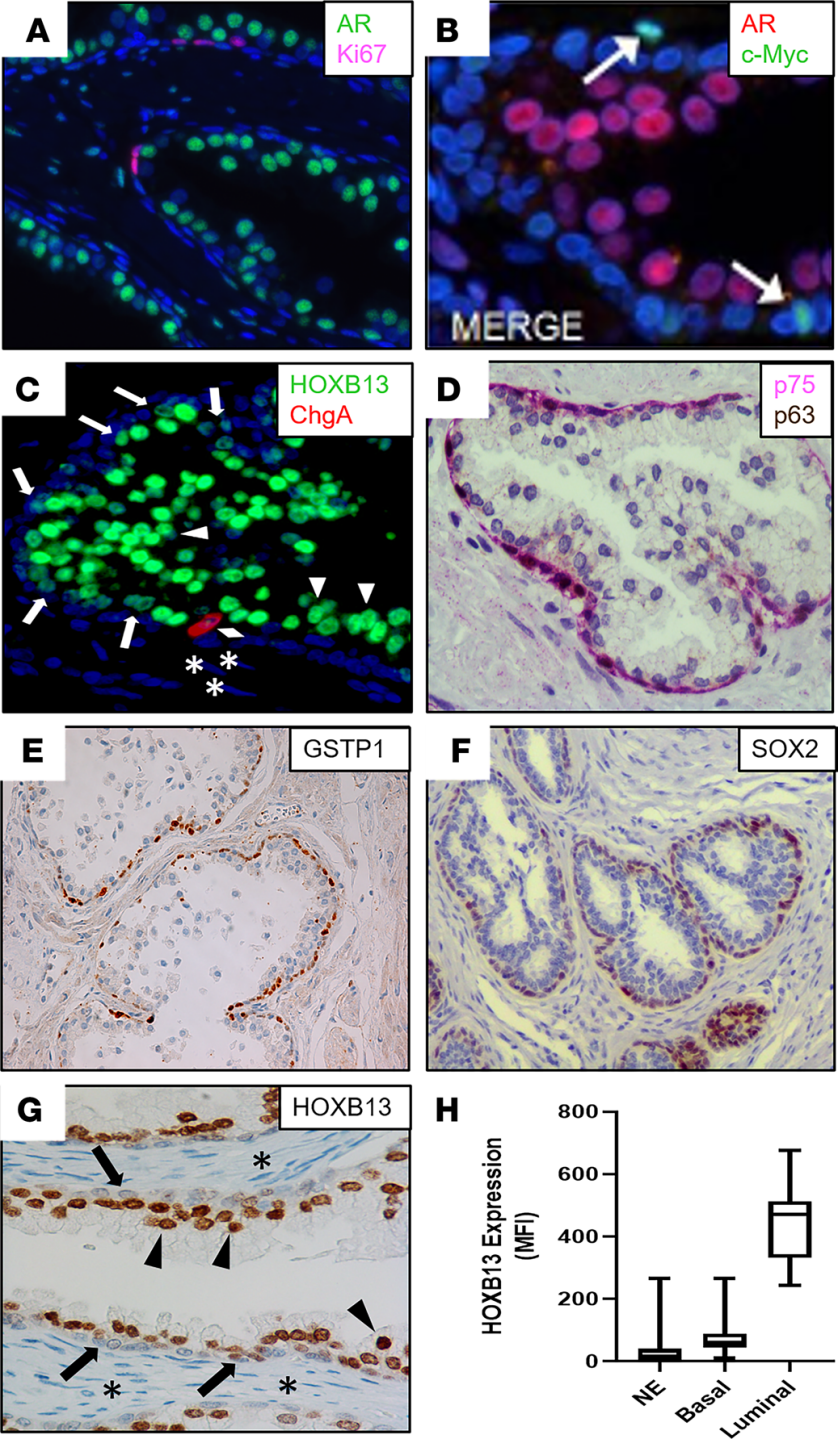
Lineage marker expression in the benign prostate. (**A**) IF staining of the AR (green) and Ki67 (pink), which documents that the majority of proliferation is restricted to the basal epithelial layer. Nuclei stained with DAPI (blue). (**B**) IF staining of c-Myc (green) and AR (red), documenting that the small subset of basal cells expressing c-Myc does not express AR. Nuclei stained with DAPI (blue). (**C**) IF staining of HOXB13 (green) and CHGA (red). Nuclei stained with DAPI (blue). Arrowheads indicate HOXB13-high luminal cells. Arrows indicate HOXB13-low basal cells. Diamond indicates CHGA^+^ neuroendocrine cell. Asterisks indicate HOXB13^–^ stromal cells. (**D**) Dual IHC staining of NFGR (pink) and p63 (brown) identifies the basal layer. (**E**) IHC staining of GSTP1 (brown) in basal layer. (**F**) IHC staining of SOX2 in basal layer. (**G**) IHC staining of HOXB13. Arrowheads indicate HOXB13-high luminal cells. Arrows indicate HOXB13-low basal cells. Asterisks indicate HOXB13^–^ stromal cells. (**H**) Box plots indicate median and IQR range for the MFI of HOXB13 staining normalized to nuclear area in neuroendocrine (*n =* 97), basal (*n =* 97), and luminal cells (*n =* 24) of the normal prostate (whiskers = min/max values). CHGA, chromogranin A; NGFR, nerve growth factor receptor.

The normal adult human prostate is composed of a simple stratified epithelium, the homeostasis of which is maintained via adult stem/progenitor cell turnover producing a steady-state, self-renewing condition (1). In the human adult man, prostate epithelial stem cells undergo asymmetric division for self-renewal while producing progenitor cells with limited proliferative ability (2). The percentage of epithelial cells proliferating per day (i.e., 0.19% ± 0.03%) is remarkably low in the human adult benign prostate, which balances the equally low percentage of epithelial cells dying per day (3). During this steady-state maintenance condition, the turnover time (i.e., the time required to renew the epithelium) is 500 ± 79 days (3). Neither stem cells nor progenitor cells express AR protein; however, they require AR-dependent paracrine factors (i.e., andromedins) from the stroma for their proliferation but not survival (4, 5). It is proposed that a rare subset (i.e., 0.59%) of adult prostate basal cells comprises the epithelial stem/progenitor cells, which coexpress the full spectrum of prostate epithelial cell markers (i.e., keratin 5 [KRT5], KRT6A, KRT8, KRT14, KRT18, and KRT19; the transcription factor p63; glutathione-*S*-transferase π [GSTP1]), but not AR (2, 6). This is supported by the fact that although the growth fraction in basal cells based upon Ki67 expression is quite low (i.e., 1.65% ± 0.12% positive), it is 12-fold higher than the growth fraction in luminal cells (i.e., 0.14% ± 0.06% positive; Figure 1A; refs. 7, 8). Consistent with this basal location for the proliferating epithelial stem/progenitor cells is the nuclear expression of c-MYC by AR^–^ cells within this compartment in benign human glands (Figure 1B).

These basal adult progenitor cells differentiate into 1 of 3 lineage progeny (6–11). In the first lineage, progenitors at a very low frequency (i.e., <1%) differentiate into proliferation-quiescent neuroendocrine (NE) cell progeny with a loss of expression of p63, GSTP1, and basal cell KRTs (KRT5, KRT6A, KRT14, and KRT19) without gaining expression of AR (9–13). Although the majority of these NE cells lack detectable expression of the highly prostate-specific transcription factor, HOXB13 (Figure 1C), a small subset (i.e., 17%) expressed this protein at a low level equal to that expressed by basal cells. In contrast, these cells acquire a high expression of NE lineage markers, such as chromogranin A (CHGA; Figure 1C), CHG B (CHGB), and synaptophysin (SYP; refs. 9–13). In the second lineage, progenitors differentiate into basal cell progeny, which mature and maintain the expression of KRT5, KRT6A, KRT14, and KRT19; p63 (Figure 1D); and GSTP1 (Figure 1E), while losing the expression of KRT8 and KRT18 without gaining the expression of AR (6–13). In addition, these basal cells characteristically express the transcription factors SOX2 (Figure 1F; ref. 14), YAP-1 (15) and nerve growth factor receptor (NGFR, also known as p75; Figure 1D; ref. 16), coupled with low-to-moderate HOXB13 expression (Figure 1G; ref. 17), consistent with its expression being AR independent (18). In the third lineage, progenitors differentiated into luminal cell progeny, which maintained the expression of KRT8 and KRT18 while losing the expression of all other stem/progenitor markers (i.e., KRT5, KRT6A, KRT14, KRT19, and p63, Figure 1D; GSTP1, Figure 1E; SOX2, Figure 1F; YAP-1 and NFGR, Figure 1D; refs. 6–11, 15). In addition, they acquire the expression of AR (6) and prostate-specific membrane antigen (PSMA, also known as FOLH1; ref. 19), along with a 6-fold increase in HOXB13 (Figure 1, G and H; refs. 17, 20). AR transcriptional activity is not required, however, for commitment to luminal cell differentiation (21) because HOXB13 is not an AR target gene (18) and PSMA transcription is inhibited by AR (22). In contrast, AR expression and ligand occupancy are required for terminal luminal differentiation into a mature proliferation-quiescent secretory cell (21). This terminal differentiation is characterized by the gain of expression of AR-dependent prostate luminal cell lineage markers, such as NKX3.1, prostate-specific antigen (PSA, also known as *KLK3*), and hK2 (*KLK2*) with no expression of NE markers (6, 23) or the proliferation markers, Ki67 (Figure 1A) or c-Myc (Figure 1B).

During prostate carcinogenesis, molecular changes occur in prostate epithelial cells such that AR signaling (ARS) is subverted from a growth suppressor of c-Myc expression to a cell autonomous oncogenic stimulator of c-Myc expression and thus malignant growth (24–26). Due to this acquired oncogenic ARS addiction, androgen deprivation therapy (ADT) is the standard of care for metastatic castration-resistant prostate cancer (mCRPC). This is because ADT not only inhibits PC cell proliferation but also induces apoptotic cell death (4). Although initially responsive to such “castration therapy,” metastatic cancer cells inevitably progress to a CR state given enough time and selective pressure (27). In the majority of cases, these mCRPC cells continue to express AR and their lethal growth is still stimulated by AR-dependent transcription despite greater than 90% suppression of serum androgen by ADT (27). These results validate that further disrupting AR function is a rational therapeutic approach for mCRPCs progressing on ADT.

Based upon this realization, next-generation ARS inhibitors (ARSis), such as abiraterone acetate (Abi), enzalutamide (Enza), and apalutamide, were developed and clinically documented to increase the survival of men with mCRPC, progressing after first-line ADT and when given in combination with first-line ADT (27). Despite these advances, mCRPC remains a lethal disease due to the inevitable progression of these cancers to an ARSi-resistant state (28, 29). Approximately one third of these ARSi-resistant cancers are AR^–^ (28). The proposed mechanism for this progression involves an initially AR^+^ adenocarcinoma (ARPC) “losing” its luminal cell differentiation via the loss of AR activity (30–34). It has been proposed that such a loss of AR-dependent transcription enables “lineage plasticity,” driving transdifferentiation of the initial ARPC to a more aggressive lethal AR^–^ treatment–related NEPC phenotype (30–35).

Therefore, to interrogate the relationship between ARSi resistance and NE differentiation, 3 complementary approaches were taken. First, the growth characteristics and expression of basal versus luminal versus NE lineage markers were evaluated in a large series (*n =* 23) of previously characterized patient-derived xenografts (PDXs) in addition to several newly established PDXs, which collectively recapitulate the full spectrum of clinically important genetic alterations in mCRPC. Second, hypermethylation of the AR promoter as a putative mechanism for the loss of AR expression was evaluated. Third, the in vitro and in vivo growth characteristics versus marker expression of the ARPC LNCaP-95 (LN-95) mCRPC cells initially exhibiting AR activity were determined following CRISPR/Cas9 dependent elimination of total AR protein expression.

## Results

### Development of ARSi-resistant NEPC does not require prior ADT.

**Figure 2:**
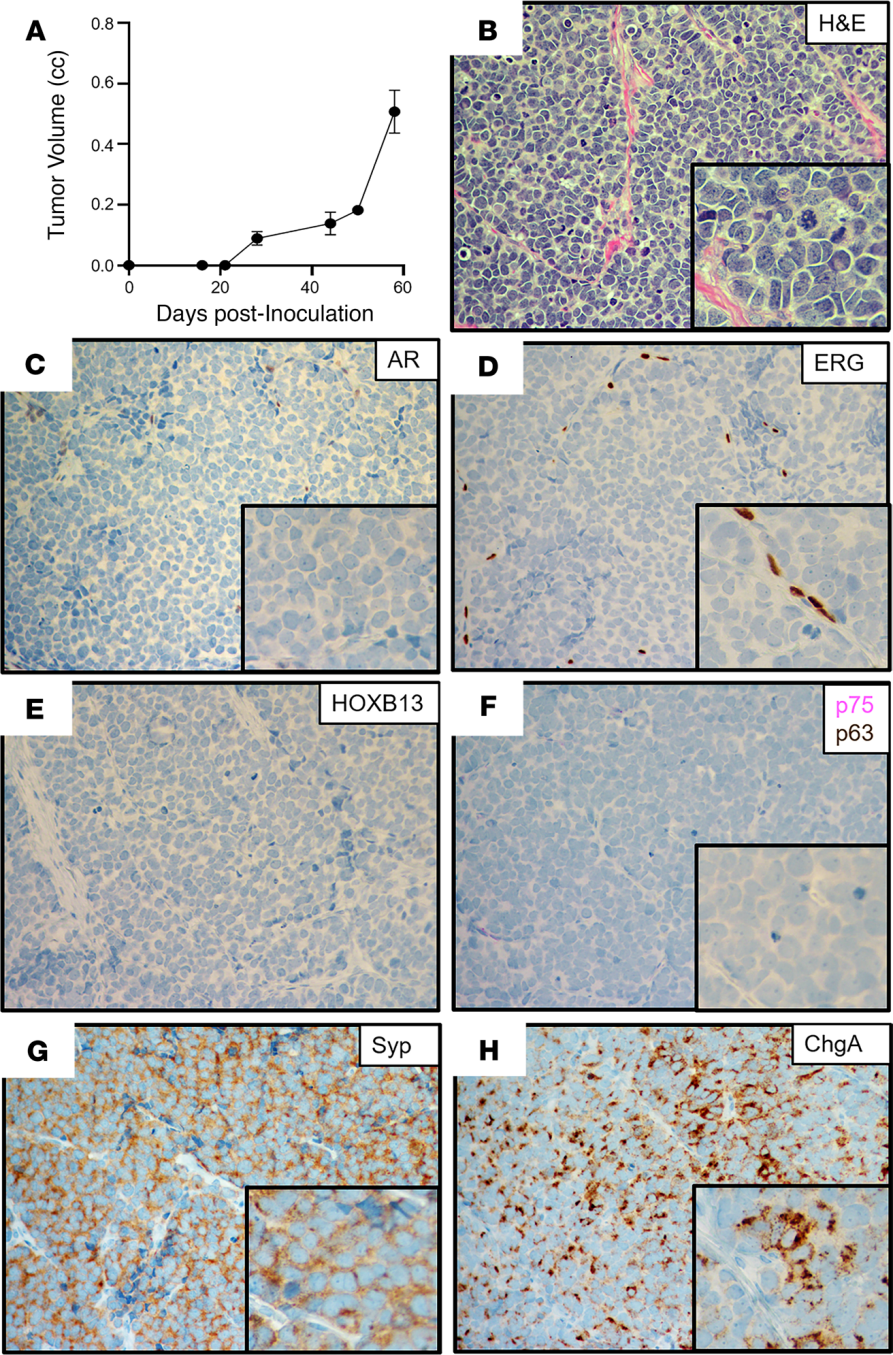
Characteristics of the NCI-H660 xenograft. (**A**) Growth rate in castrated NSG male mice (*n =* 5). (**B**) H&E histology (original magnification, ×200; inset [original magnification, ×400]). (**C**–**H**) IHC (original magnification, ×200) for (**C**) AR; (**D**) ERG (note positive staining in tumor endothelial cells); (**E**) HOXB13; (**F**) NFGR (pink) and p63 (brown) dual stain; (**G**) SYP; and (**H**) CHGA. NGFR, nerve growth factor receptor; SYP, synaptophysin; CHGA, chromogranin A.

The NCI-H660 cell line was established from a cervical lymph node metastasis from an untreated 63-year-old man diagnosed with small cell cancer of the prostate who presented with metastatic sites in the brain, liver, lymph nodes, subcutaneous tissue, bone, and bone marrow (36). The patient died 4 days after tissue harvest (i.e., 18 days after initial diagnosis) having never received ADT. The in vivo growth of H660 cells in adult male NSG hosts is ARSi resistant, because it grew equally well in intact or castrated hosts with a doubling time of 10 ± 5 days (Figure 2A), which was not affected by daily oral treatment with a therapeutically effective dose of either Abi or Enza. Histologically, this xenograft has been classified as a small-cell carcinoma (Figure 2B). It has a *TP53* exon 9–11 deletion (37) and *TMPRSS2–ERG* fusion due to a homozygous intronic deletion (38), but expressed neither AR (Figure 2C) nor ERG (Figure 2D). It also did not express HOXB13 (Figure 2E; ref. 39), the basal cell markers GSTP1, KRT5, NGFR (Figure 2F), or p63 (Figure 2F). It did, however, uniformly express NE markers such as SYP (Figure 2G) and CHGA (Figure 2H). Thus, H660 is a “classic” ARSi-resistant NEPC, which developed without ADT treatment.

### AR does not suppress NE differentiation in amphicrine PC PDXs.

**Figure 3:**
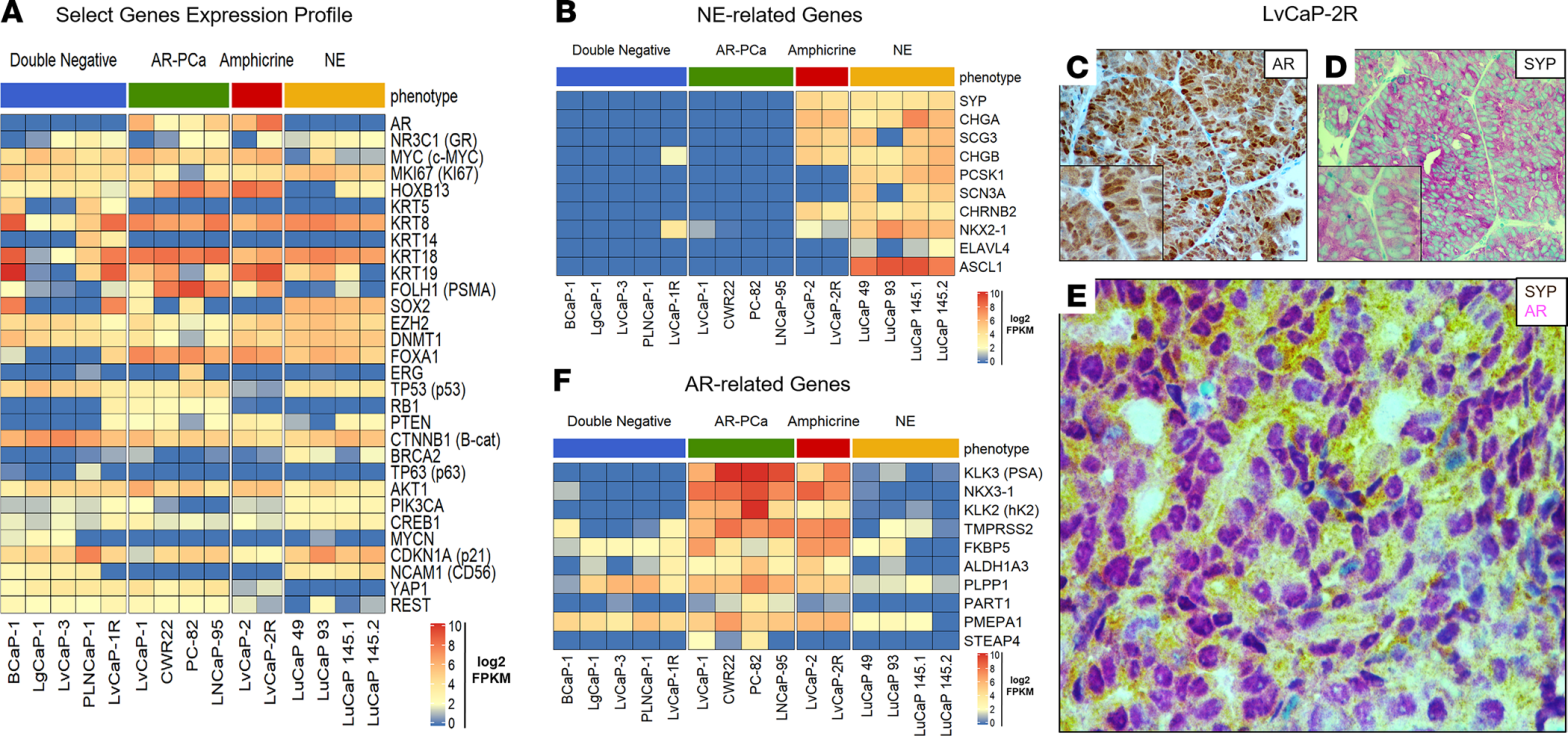
Gene expression in lethal mCRPC PDXs and amphicrine phenotype in LvCaP-2R. Lethal mCRPC PDXs analyzed for RNA expression of (**A**) select genes and (**B**) NE-associated genes. (**C**–**E**) IHC step-section of LvCaP-2R PDX stained for (**C**) AR (original magnification, ×200), (**D**) SYP (original magnification, ×200), and (**E**) dual staining (original magnification, ×400) for AR (pink) and SYP (brown), documenting the coexpression of both markers in the same cell (i.e., amphicrine). (**F**) RNA-Seq analysis for AR-regulated genes in a panel of PDXs representing different phenotypes (e.g., DN, ARPC, amphicrine, and NE). mCRPC, metastatic castration-resistant prostate cancer; NE, neuroendocrine; PDXs, patient-derived xenografts; SYP, synaptophysin; DN, double-negative.

LvCaP-2 is a newly described PC PDX derived from a liver metastasis obtained at rapid autopsy at John Hopkins from an ARSi-resistant patient with mCRPC (29). When adult male hosts bearing the LvCaP-2 PDX are castrated, the cancer stops growing for approximately 1 month before relapsing (29). Subsequent passage of a relapsing tumor in castrated hosts results in a variant, named LvCaP-2R, that grows equally well in intact and castrated hosts (doubling time of 10 ± 3 days versus 9 ± 2 days, respectively). The growth of LvCaP-2R in castrated mice is resistant to daily oral treatment with Abi or Enza (29). Histologically, LvCaP-2 and LvCaP-2R are high-grade adenocarcinomas, which genetically have a hemizygous loss-of-function (LOF) truncating mutation in *TP53* (T211fs) and hemizygous deleterious mutation (R130Q) in *PTEN* with a loss of PTEN protein expression (29). Although they have WT *RB1*, there is only limited focal expression of RB1 protein. In addition to expressing prostate-specific HOXB13 and luminal-specific, but not basal-specific, markers (Figure 3A), they expressed NE markers (Figure 3B). This is despite expressing AR at a 52-fold higher mRNA level (Figure 3A) with 11-fold higher nuclear localization of AR protein compared with normal prostate luminal cells (29). Importantly, AR and NE markers like SYP are coexpressed in the same cell (Figure 3, C–E). This AR is functional as documented by the expression (Figure 3F) and secretion of AR target proteins such as PSA (Supplemental Figure 1A; supplemental material available online with this article; https://doi.org/10.1172/jci.insight.146827DS1). They also expressed *REST* and *YAP1* (Figure 3A), the latter of which is a basal lineage marker, and neither of which is expressed in NEPC (15, 40). These cancers thus represent “amphicrine” prostate carcinomas (AMPCs; i.e., AR^+^/NE^+^; refs. 41, 42). A similar coexpression of luminal and NE markers without basal marker expression occurs in the AR^+^ LuCaP-77CR PDX, which is an ARSi-resistant AMPC variant of LuCaP-77 derived from a bone (femur) metastasis at rapid autopsy (43). These results document that ARS does not suppress NE differentiation in ARSi-resistant AR^+^/NE^+^ double-positive AMPCs and that NE differentiation can occur in the presence of ARSi-resistant AR signaling, resulting in ARSi-resistant AR^+^/NE^+^ double-positive AMPCs.

### ARSi resistance in PDXs lacking AR expression does not necessitate NE differentiation.

**Figure 4:**
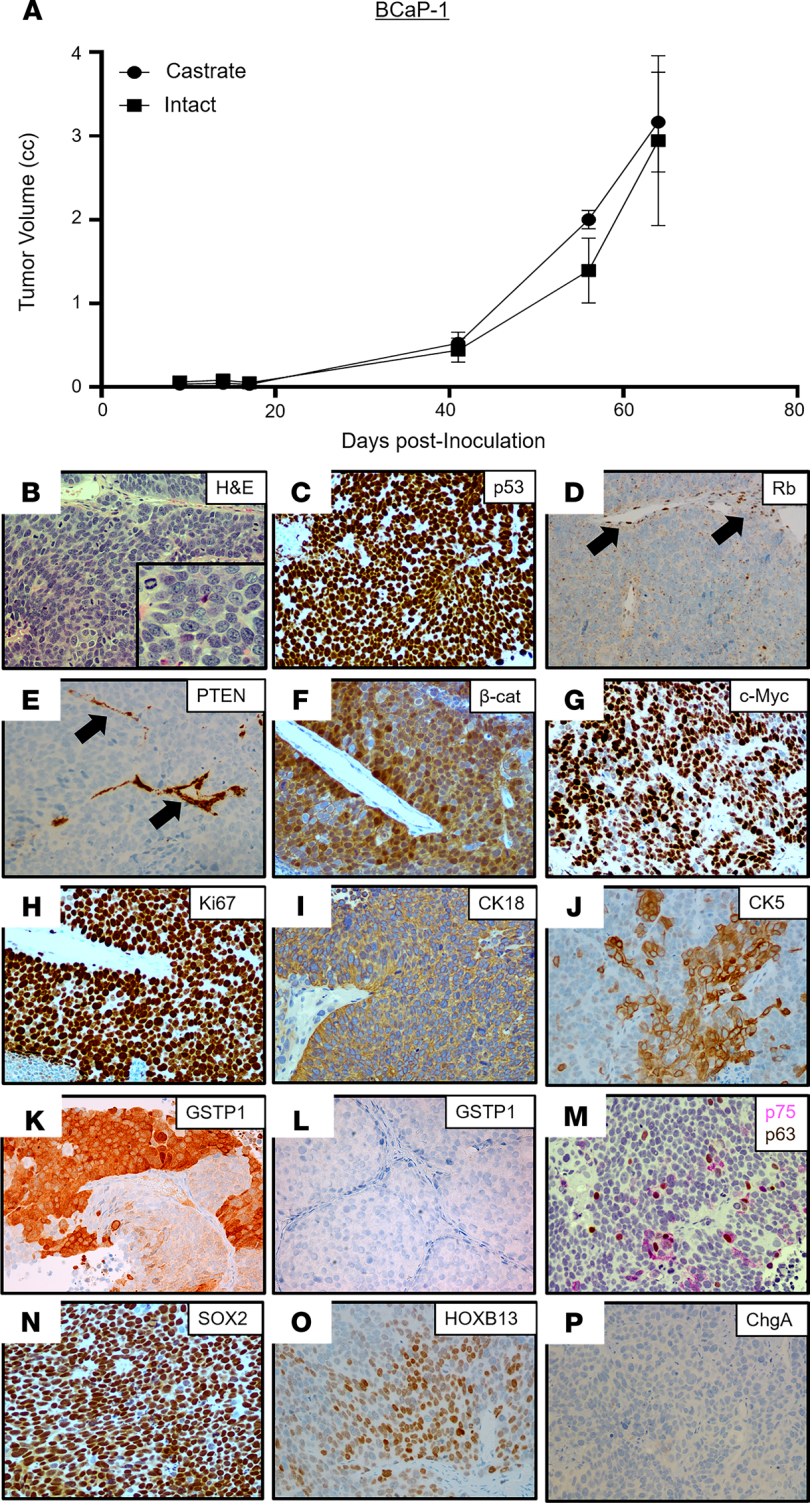
Characteristics of the BCaP-1 PDX. (**A**) Growth rate in intact vs. castrated NSG male mice (*n =* 5). (**B**) H&E histology (original magnification, ×200; inset [original magnification, ×400]). (**C**–**P**) IHC (original magnification, ×200) for (**C**) p53; (**D**) Rb (note that endothelial cell nuclei are an internal positive control for staining [black arrows]); (**E**) PTEN (note that endothelial cells are an internal positive control for staining [black arrows]); (**F**) β-catenin; (**G**) c-MYC; (**H**) Ki67; (**I**) CK18; (**J**) focal CK5; (**K**) GSTP1 in BCaP-1 (positive); (**L**) GSTP1 in SkCaP-1 (negative control); (**M**) dual staining for p75 (pink) and p63 (brown); (**N**) Sox2; (**O**) HOXB13; and (**P**) CHGA. PDX, patient-derived xenograft; CHGA, chromogranin A.

The BCaP-1 PDX was derived from a soft tissue metastasis adjacent to the right tibia obtained at rapid autopsy from a 63-year-old African American patient, who, at the time of initial presentation, had bone and lymph node metastases and an initial diagnostic prostate biopsy that was positive for carcinoma with a Gleason sum score of 9. Over the next year, the patient was treated with ADT followed by palliative external beam radiation of the bone before death but never received treatment with next-generation ARSis (Table 1 and Supplemental Figure 2A). At autopsy, 3 of the 4 metastatic lesions collected were completely negative for AR, PSA, and NKX3.1, including the bone metastasis used to establish the BCaP-1 PDX (Supplemental Table 1). In 1 of the collected metastases (i.e., bone — L4) and the localized prostate lesion, AR staining was heterogeneous (Supplemental Table 1). The expression of AR-dependent genes (PSA, NKX3.1, etc.) was consistent with the AR expression pattern in these lesions. In contrast, all 4 of the metastatic lesions collected from the patient in addition to the localized cancer in the prostate had *PTEN*-loss, *RB1*-loss, and mutated *TP53* (i.e., genetic drivers), which are consistent with the PDX. Despite the patient never being exposed to ARSi treatment, the in vivo growth of BCaP-1 PDX is ARSi resistant because it grows equally well in intact or castrated hosts, with a doubling time of 20 ± 5 days (Figure 4A), which was not affected by additional treatment with either Abi or Enza. This is consistent with the fact that the metastatic lesion from which this PDX is derived was AR^–^. Histologically, this PDX is a high-grade carcinoma (Figure 4B). Based upon a combination of RNA-Seq, Western blotting, IHC staining, and targeted DNA sequencing analyses, Table 1 summarizes the most relevant characteristics of the BCaP-1 PDX. Consistent with the metastatic lesions in the patient, BCaP-1 uniformly expressed mutated *TP53* (R175H; Figure 3A and Figure 4C) coupled with minimal expression of a mutated *RB1* (P298fs) allele and loss of the other WT *RB1* allele (Figure 3A and Figure 4D). These cells uniformly lacked *PTEN* expression due to a homozygous deletion (Figure 3A), resulting in no detectable PTEN protein (Figure 4E). They also expressed a mutated *CTNNB1* (S45F; Figure 3A), which was localized in the nucleus (Figure 4F), presumably activating CTNNB1-driven gene expression. BCaP-1 had a high expression of *c-MYC* and *Ki67* (Figure 3A), with a high proportion of cells showing nuclear localization of these proteins (Figure 4, G and H). This is despite minimal expression of *AR* or glucocorticoid receptor (*GR*) mRNA (Figure 3A) and no expression of AR or GR protein. Consistent with the lack of AR protein, BCaP-1 did not express AR target genes like *PSA* and *NKX3-1* that are characteristic of luminal cells (Table 1, Figure 3E, and Supplemental Figure 1A). However, they did uniformly express other markers characteristic of luminal cells, such as *KRT8* and *KRT18* (Figure 3A and Figure 4I), while heterogeneously expressing characteristic basal cell markers, such as *KRT5* (Figure 3A and Figure 4J), GSTP1 (Figure 4K; SkCaP-1 shown in Figure 4L as a negative control for GSTP1 staining), NGFR (also known as p75 neurotrophin receptor; Figure 4M), and p63 (Figure 4M), coupled with a uniform expression of SOX2 (Figure 4N; refs. 14, 44, 45). Additionally, they heterogeneously expressed a moderate-to-high level of nuclear HOXB13 protein (Figure 4O). The retention of HOXB13 expression confirms its prostatic origin. This moderate-to-high level of HOXB13 expression in a subset of cells is significant because this is the level of nuclear expression characteristic of prostatic luminal cells (20) and is consistent with its expression being AR-independent (18). These cells also expressed *FOXA1* (Figure 3A), which is an important coregulator of *HOXB13* via its binding to a 37-bp regulatory element that activates the expression independent of AR transcriptional activity (18). This heterogeneous moderate-to-high expression is consistent with why this PDX had a lower level of *HOXB13* mRNA (Figure 3A) and protein detected by Western blot (Supplemental Figure 1B than ARPCs. BCaP-1 lacked the expression of *ERG* (Figure 3A) and the majority of NE-related genes (Figure 3B), including CHGA (Figure 4P). They also expressed *REST* (Figure 3A) and the basal marker *YAP1* (Figure 3A), neither of which are expressed in NEPC (15, 40). Collectively, these results document that, despite the lack of AR expression by BCaP-1, even when coupled with LOF mutations in *PTEN* and *RB1*, plus a putative gain-of-function mutation in *TP53* together with the overexpression of *DNMT1* and *EZH2* (Figure 3A), these genetic change do not drive BCaP-1’s differentiation into a NEPC. Rather, it is an example of an AR^–^/NE^–^ double-negative (DN) PC (DNPC) heterogeneously composed of cells expressing both basal and luminal cell characteristics, suggestive that their cancer-initiating cell was an AR^–^ progenitor cell whose malignant transformation did not require exposure to ARSis.

**Figure 5:**
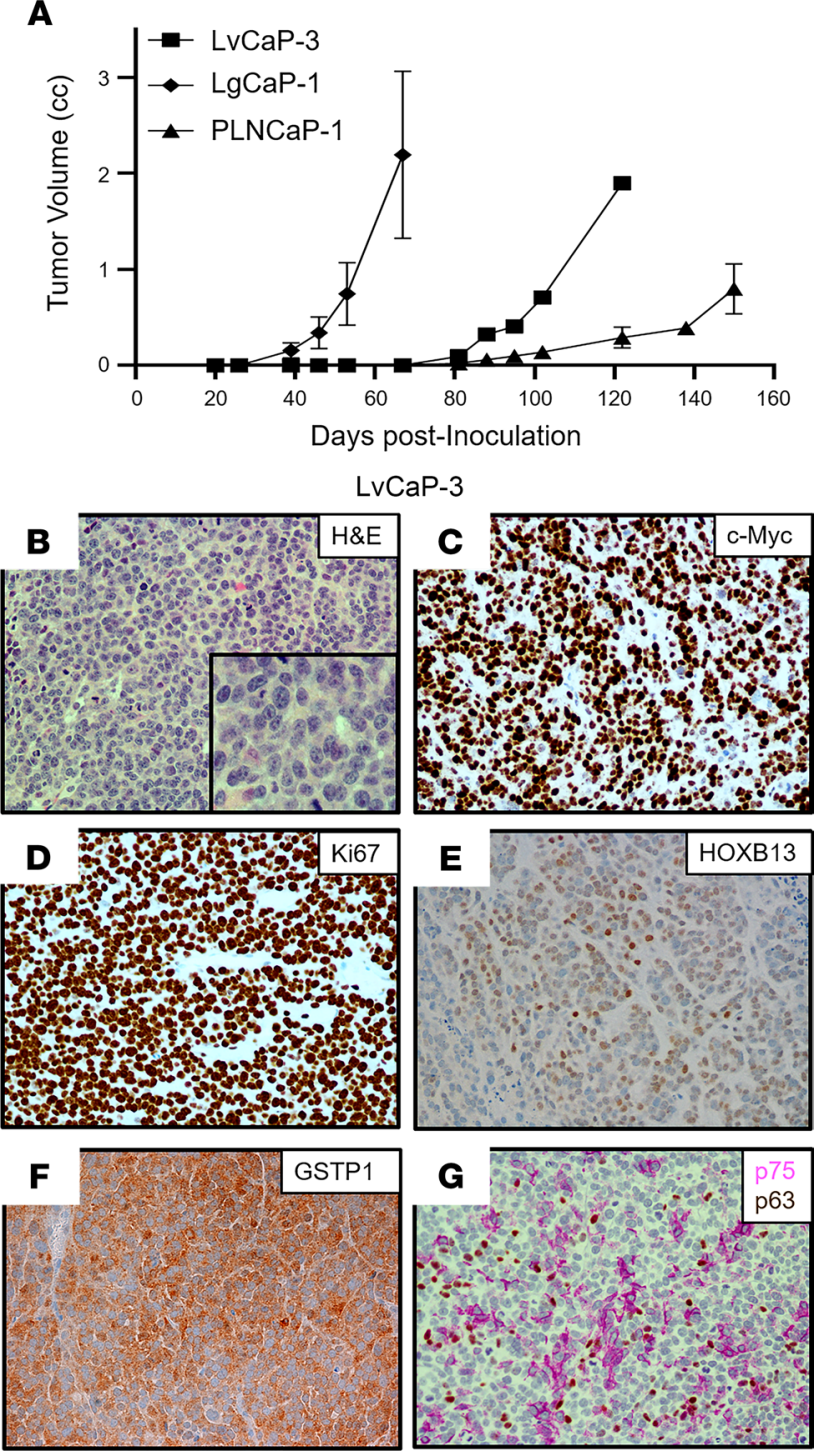
Characteristics of the LvCaP-3 PDX. (**A**) Comparative growth rate in castrated NSG male mice (*n =* 5) of LvCaP-3, LgCaP-1, and PLNCaP-1. (**B**) H&E histology (original magnification, ×200; inset [original magnification, ×400]). (**C**–**F**) IHC (original magnification, ×200) for (**C**) c-MYC, (**D**) Ki67, (**E**) HOXB13, and (**F**) GSTP1. (**G**) Dual staining for NGFR (pink) and p63 (brown). PDX, patient-derived xenograft; NGFR, nerve growth factor receptor.

**Figure 6:**
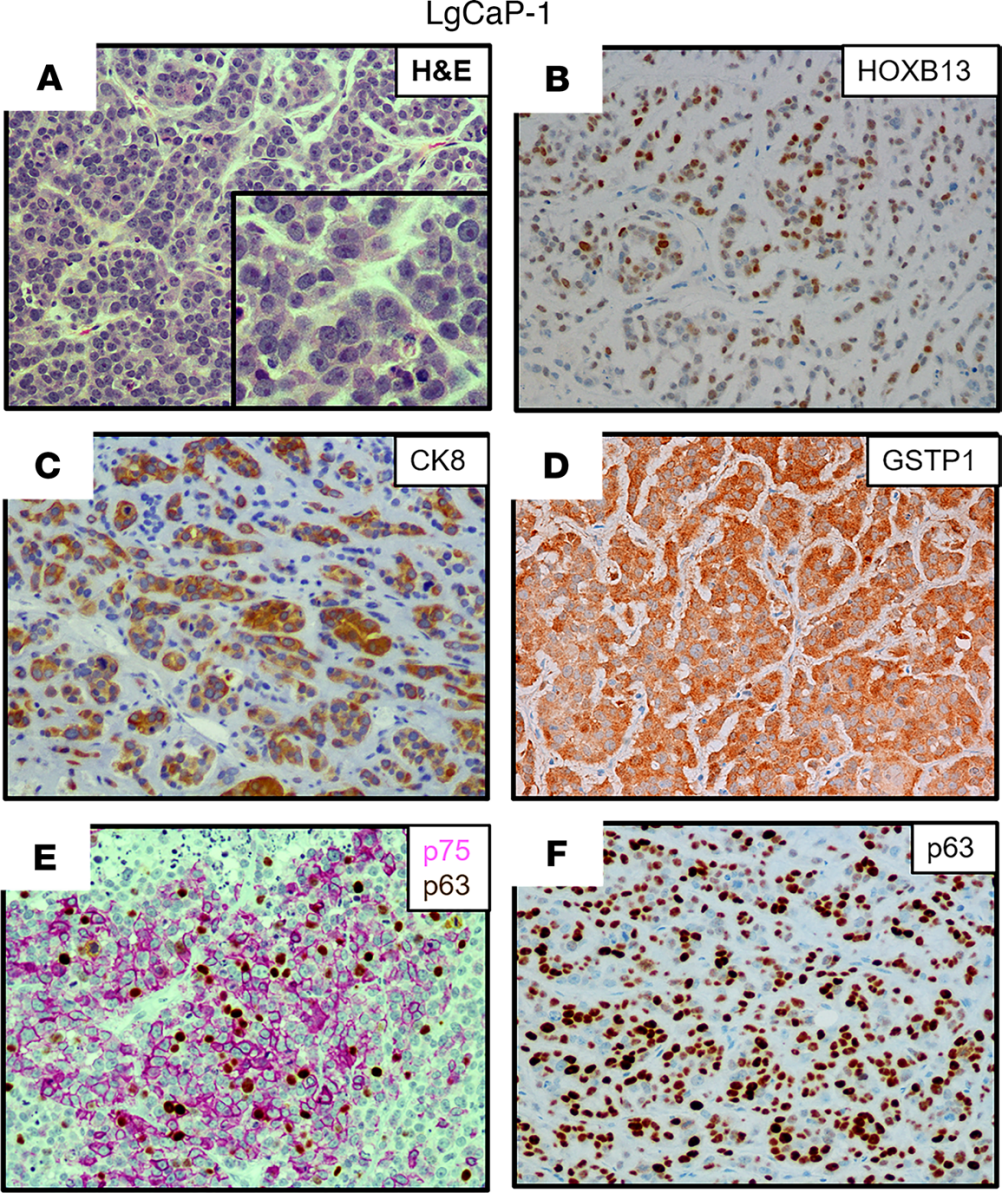
Characteristics of the LgCaP-1 PDX. (**A**) H&E histology (original magnification, ×200; inset [original magnification, ×400]); (**B**–**E)** IHC (original magnification, ×200) for (**B**) HOXB13; (**C**) CK8; (**D**) GSTP1; (**E**) NGFR (pink); and (**F**) p63 (brown). PDX, patient-derived xenograft; NGFR, nerve growth factor receptor.

**Figure 7:**
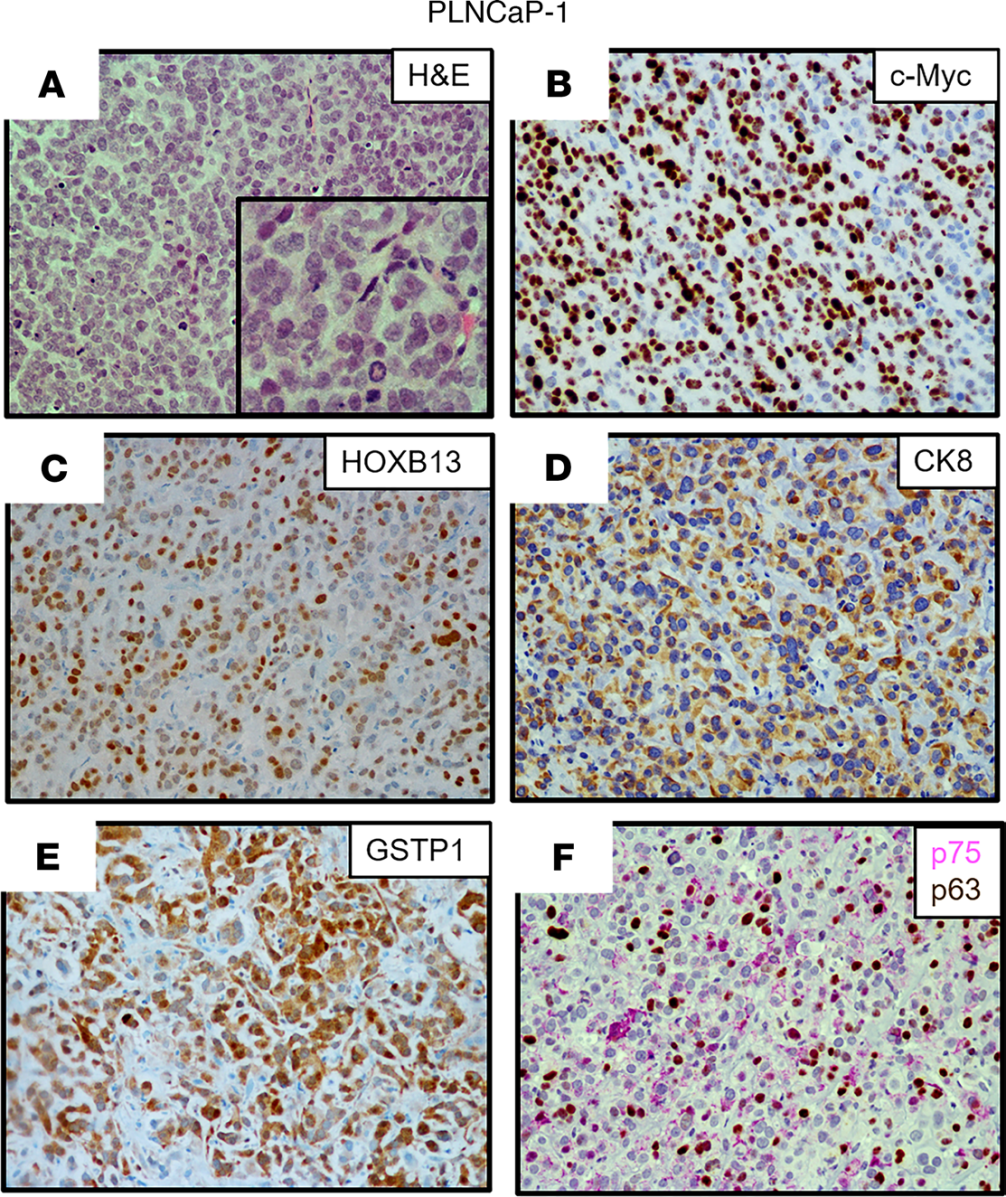
Characteristics of the PLNCaP-1 PDX. (**A**) H&E histology (original magnification, ×200; inset [original magnification, ×400]). (**B**–**F**) IHC (original magnification, ×200) for (**B**) c-MYC; (**C**) HOXB13; (**D**) CK8; (**E**) GSTP1; and (**F**) NGFR (pink) and p63 (brown). PDX, patient-derived xenograft; NGFR, nerve growth factor receptor.

Three additional PDXs were established from a 65-year-old European American patient with a germline *BRCA2* mutation (Y2215fs) who underwent resection for a noninvasive urothelial carcinoma of the bladder a year before having a prostate biopsy that was positive for Gleason 9 PC with perineural invasion. Over the next 3 years, the patient had a radical prostatectomy for locally advanced disease (i.e., extraprostatic extension, seminal vesicle invasion, lymphatic invasion), followed by ADT, external beam radiation, taxane chemotherapy, and olaparib before undergoing a rapid autopsy upon his death (Supplemental Figure 2B). All metastatic lesions collected from this patient had *PTEN*-loss and *RB1*-loss, in addition to mutated *TP53* (Supplemental Table 1). From this autopsy, 3 independent PDXs were established from a liver metastasis (LvCaP-3), a lung metastasis (LgCaP-1), and a peripancreatic lymph node metastasis (PLNCaP-1). The in vivo growth of each of these PDXs was ARSi resistant, as documented by the fact that each grew equally well in intact versus castrate hosts and was not affected by the addition of treatment with either Abi or Enza. Interestingly, though derived from the same patient with lethal mCRPC, these 3 ARSi-resistant PDXs had different in vivo growth rates (Table 1 and Figure 5A), despite the fact that all 3 PDXs are histologically high-grade carcinomas (Figure 5B, Figure 6A, and Figure 7A).

Table 1 summarizes the most relevant characteristics of these 3 additional ARSi-resistant PDX models. All 3 of these additional PDXs lacked the expression of *ERG* (Figure 3A) and NE markers, including *CHGA*, *CHGB*, and *SYP* (Figure 3B), with minimal expression of either mutated *BRCA2* (Y2215fs) (Figure 3A) or mutated *TP53* (R282W), and no expression of *PTEN* due to homozygous deletion. In addition, they minimally expressed *RB1* mRNA with no detectable nuclear expression of RB1 protein (Figure 3A and Table 1). None of the cancer cells in these 3 PDXs express AR protein, thus explaining why they are resistant to ARSis. The tumors had a high proportion (>50%) of cells with nuclear staining for c-MYC (Table 1, Figure 5C, and Figure 7B) and Ki67 (Table 1 and Figure 5D). They did not express *KLK3* (PSA), *FOLH1* (PSMA), or *NKX3.1* (Table 1, Figure 3, A and E, and Supplemental Figure 1A). Again, similar to AR^–^ BCaP-1 cells, the fact that there was no expression of PSA or NKX3.1 in LvCaP-3, LgCaP-1, or PLNCaP-1 cells is predictable because these are known AR target genes. This is despite the fact that these cells expressed *GR* (Figure 3A). All 3 heterogeneously expressed a moderate-to-high level of nuclear HOXB13 protein (Figure 5E, Figure 6B, and Figure 7C). Again, this cellular heterogeneity is consistent with these PDXs having a lower level of *HOXB13* mRNA (Figure 3A) and protein detected by Western blot (Supplemental Figure 1B) than ARPCs. The retention of HOXB13 expression in all 3 of these PDXs confirms their prostatic origin. Unlike BCaP-1 cells, however, HOXB13 expression in all 3 of these latter PDXs was independent of *FOXA1* because they essentially had no expression of this transcription factor (Figure 3A).

All 3 of these PDXs retained the expression of luminal characteristic *KRT8* (Figure 3A, Figure 6C, and Figure 7D) and *KRT18* (Figure 3A). Interestingly, although neither LvCaP-3 nor LgCaP-1 express basal characteristic *KRT5* or *KRT14*, PLNCaP-1 coexpressed *KRT5*, *KRT8, KRT14*, *KRT18*, and *KRT19* (Figure 3A). They also all uniformly expressed the basal marker GSTP1 (Figure 5F, Figure 6D, and Figure 7E), in addition to other basal characteristic markers such as NGFR focally and p63 sporadically (Figure 5G, Figure 6, E and F, and Figure 7F). Thus, despite the lack of AR and RB1 expression coupled with mutations in *PTEN* and *TP53* and the overexpression of *DNMT1* and *EZH2* (Figure 3A), they were not NEPCs. Again, they expressed *REST* and the basal marker *YAP1* (Figure 3A), which are not expressed in NEPC (15, 40). Thus, these PDXs are again examples of AR^–^/NE^–^ DNPCs with heterogeneous basal and luminal cell characteristics.

### Progression of ARPC PDX to ARSi resistance.

These PDX models document that the lack of AR-dependent transcription in AR^–^ PC cells does not necessitate differentiation into NEPC, even when combined with LOF/expression of PTEN, RB1, and p53. A possible explanation is that the cancer-initiating cells in these DN-PDXs are derived from transformed progenitor cells that never expressed AR and thus are unresponsive to AR-targeted therapy. Clearly, however, the majority of mCRPCs express AR. This raises the issue of whether the subset of ARPCs that lose AR expression in their progression to ARSi resistance induces lineage transdifferentiation into NEPC.

**Figure 8:**
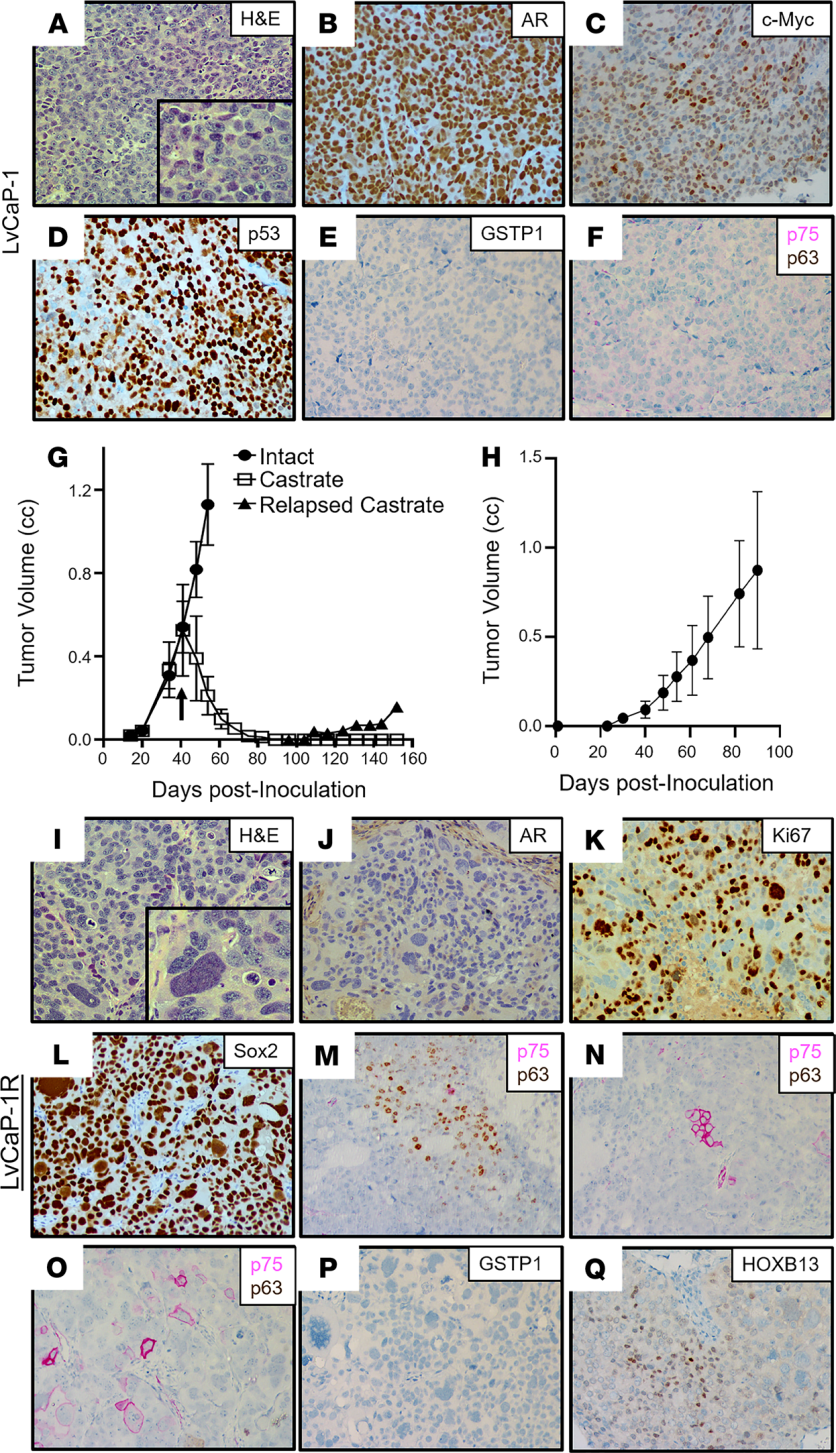
Characteristics of the LvCaP-1 PDX. (**A**) H&E histology (original magnification, ×200; inset [original magnification, ×400]). **(B**–**F**) IHC (original magnification, ×200) for (**B**) AR; (**C**) HOXB13; (**D**) p53; (**E**) GSTP1; and (**F**) dual staining for NGFR (pink) and p63 (brown). (**G**) Growth rate in intact and subsequent regression and relapse to castration in NSG male mice (*n =* 5 each). Characteristics of the LvCaP-1R PDX. (**H**) Growth rate in castrated NSG male mice (*n =* 5 each); (**I**) H&E histology (original magnification, ×200; inset [original magnification, ×400]) showing a pleomorphic giant cell. (**J**–**Q**) IHC (original magnification, ×200) for (**J**) AR; (**K**) Ki67; (**L**) SOX2; (**M**–**O**) dual staining for NGFR (pink) and p63 (brown); (**P**) GSTP1; and (**Q**) HOXB13. PDX, patient-derived xenograft; NGFR, nerve growth factor receptor.

To test this possibility, another newly derived PDX, LvCaP-1, was used as a model system. LvCaP-1 was derived from a liver metastasis obtained at rapid autopsy from a 64-year-old European American patient who was treated over a 17-year period starting with a radical prostatectomy (Gleason sum 8) and then a PC vaccine (GVAX), followed by ADT, docetaxel plus strontium-89, external beam radiation, Abi, etoposide, and cisplatin (Supplemental Figure 2C; ref. 46). Similar to the radical prostatectomy specimen, all metastatic lesions collected from this patient at the time of autopsy were AR^+^ and NKX3-1^+^, in addition to having *PTEN*-loss and mutated *TP53* (Supplemental Table 1). The most relevant characteristics of this PDX are summarized in Table 1. Histologically, LvCaP-1 is a high-grade adenocarcinoma (Figure 8A). Similar to the original patient-derived liver metastasis (46), this PDX had a 20-fold amplification of the *AR* gene locus and a 64-fold higher level of WT *AR* mRNA compared with localized PC (Figure 3A). Essentially, all LvCaP-1 cells exhibited high nuclear staining of AR-FL (Figure 8B). A high proportion (>70%) of cells expressed *c-MYC* and *Ki67* (Figure 3A). LvCaP-1 expressed *NKX3-1*, *HOXB13 (*mutated *G84E)*, *FOLH1*, *KLK2*, and *KLK3* (Figure 3, A and E, and Figure 8C), but with only a low level of PSA secretion (i.e., serum PSA of 1.4 ± 0.4 ng/mL/g tumor; Supplemental Figure 1A). This is coupled with the expression of mutated *TP53* (R248Q; Figure 8D), *SOX2*, mutated *SPOP* (F133L), and mutated *PTEN* (V317fs), but with no expression of NE markers like *CHGA*, *CHGB*, and *SYP* (Figure 3, A and B). LvCaP-1 also did not express basal cell markers like GSTP1 (Figure 8E), p75 (Figure 8F), or p63 (Figure 8F).

When intact mice bearing LvCaP-1 PDXs growing with a 12 ± 2 day doubling time were castrated, the cancers regressed by greater than 90% over the next 80 days before relapsing (Figure 8G), documenting that LvCaP-1 is an androgen-responsive ARPC. A relapsing tumor was serially passaged in castrated male mice to produce the LvCaP-1R PDX, which grows with a doubling time of 15 ± 5 days in castrated hosts (Figure 8H). Oral treatment with effective daily doses of either Abi or Enza (29) has no effect upon the growth of LvCap-1R in castrated male hosts, documenting its ARSi resistance. Histologically, the LvCaP-1R is a high-grade carcinoma with focal pleomorphic giant cell features (Figure 8I). LvCaP-1R retains a 20-fold amplification of the *AR* gene. Despite this amplification, LvCaP-1R had minimal expression of *AR* mRNA (Figure 3A) and no detectable AR protein (Figure 8J). This PDX also lacked detectable expression of AR-dependent NXK3.1 and PSA, but retained a large proportion of cells expressing c-MYC and Ki67 (Figure 3A and Figure 8K). Like the parental LvCaP-1 PDX, it also retained mutated *TP53* (R248Q), mutated *PTEN* (V317fs), mutated *SPOP* (F133L), and mutated *ATRX* (Table 1 and Figure 3A) and a uniform expression of basal marker SOX2 (Figure 8L). In addition, there was no gain of NE marker expression, including *CHGA*, *CHGB*, and *SYP* (Table 1 and Figure 3B). However, it did gain heterogeneous expression of basal markers like KRT5, KRT14, p63 (Figure 8M), and NGFR (Figure 8, N and O), but not GSTP1 (Figure 8P). Importantly, it retained heterogeneous moderate-to-high expression of both the AR-independent *HOXB13* (Figure 8Q) and the *FOLH1* genes (Figure 3, A and E, and Supplemental Figure 1B), which are characteristic of luminal cells. These results document that the lack of AR expression by ARSi-resistant LVCaP-1R resulted in the acquisition of an AR^–^/NE^–^ DN phenotype with heterogeneous expression of both basal and luminal cell markers, but lacked NE differentiation.

### Response to acute loss of AR by ARSi-resistant PC cells.

**Figure 9:**
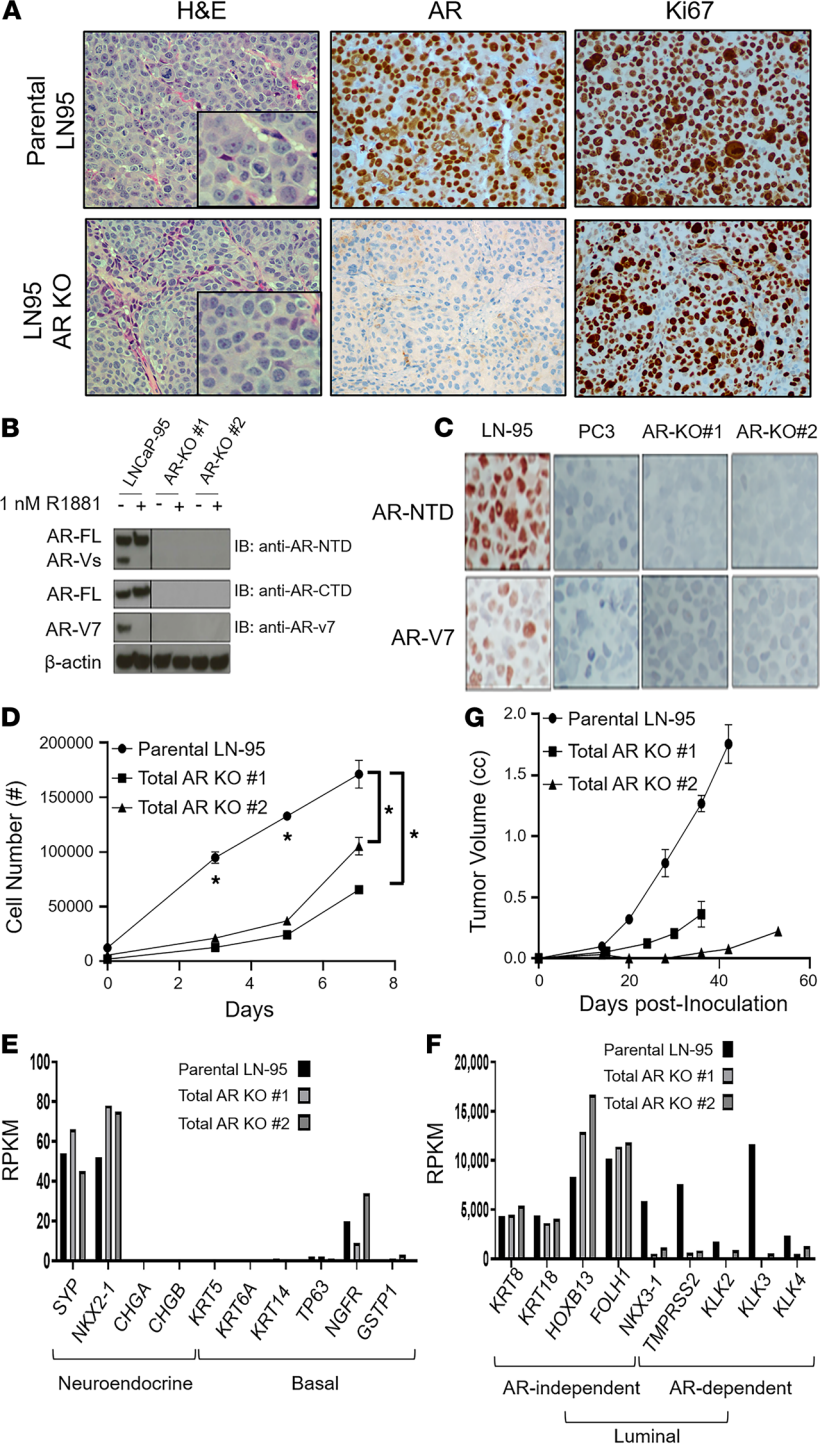
Characterization of LN-95 parental vs. **total AR-KO cells**. (**A**) Left panels are the histology (original magnification, ×200; inset [original magnification, ×400]); middle panels are the AR protein expression (original magnification, ×200); and right panels are the Ki67 expression (original magnification, ×200) of the PDXs. (**B**) Western blot documentation of the successful KO of AR protein in multiple clones of LN-95 cells. (**C**) IHC (original magnification, ×200) staining of parental LN-95 cells expressing both full-length AR (AR-FL) and AR variant 7 (AR-V7) vs. AR^–^ PC-3 cells and the AR-KO clones using an N-terminal AR antibody and an AR-V7–specific antibody. (**D**) In vitro growth of the parental LN-95 cells vs. total AR-KO clones in 10% CS-FBS media, with asterisks denoting a significant difference at the *P <* 0.05 level. (**E**) RNA-Seq–based comparison of the expression of NE-specific and basal-specific genes in total AR-KO clones compared with parental LN-95 cells. (**F**) RNA-Seq–based comparison of the expression of AR-independent and AR-dependent luminal-specific genes in total AR-KO clones compared with parental LN-95 cells (note the significant difference in the magnitude of the y axis between panels). (**G**) In vivo growth of the total AR-KO clones vs. the parental LN-95 in castrated hosts. LN-95, LNCaP-95.

To directly test whether the acute loss of ARS can drive NE transdifferentiation of ARSi-resistant ARPC cells, a molecular approach was taken using the ARSi-resistant LN-95 PC cell line as a model. This cell line is a variant of LNCaP, produced by long-term in vitro growth in charcoal-stripped FBS (CS-FBS) media containing a low level of androgen (47). ARSi resistance is documented by the fact that both its in vitro and in vivo growth are resistant to Enza (29). This in vivo ARSi resistance is not due to intratumoral synthesis of androgens, as documented by the fact that serial passaging of LN-95 in castrated NSG hosts results in equivalent levels of intratumoral androgen as seen in patients treated with ARSi (i.e., Abi/Enza; refs. 48, 49). Importantly, we have documented previously that such ARSi resistance is not associated with a NE morphology, but that LN-95 remains a poorly differentiated ARPC whose growth (i.e., high Ki67 positivity) is dependent upon AR expression and signaling even when grown in a castrated host (Figure 9A, top; ref. 29).

Based upon this validation, CRISPR/Cas9 editing was used to delete the AR in LN-95 cells. Multiple clones were obtained in which both full-length and AR-V7 were simultaneously knocked out (i.e., total AR KO; Figure 9, B and C). The in vitro growth of these total AR-KO clones was slower than the parental AR-expressing LN-95 cells in CS-FBS media (Figure 9D). Importantly, these AR-KO clones did not acquire NE (i.e., dendritic) morphology in vitro (Supplemental Figure 3), nor did they upregulate NE markers like *SYP, CHGA,* or *CHGB* (Figure 9E) or gain the expression of basal markers like *KRT5, KRT14, TP63, NGFR,* or *GSTP1* (Figure 9E). They did, however, downregulate the expression of AR target genes like *NKX3-1, TMPRSS2, KLK2*, and *KLK3* (Figure 9F and Supplemental Figure 1C). Despite the loss of AR expression, they retained a high proportion of Ki67^+^ cells while remaining a high-grade carcinoma histologically in vivo (Figure 9A, bottom). These AR-KO clones, however, grew at a rate that was significantly slower (*P <* 0.05) than the parental AR-expressing LN-95 cells in castrated male hosts (Figure 9G). Importantly, the AR-KO clones retained the same high expression of AR-independent luminal genes (e.g., *KRT8, KRT18, HOXB13, FOLH1*) as the parental LN-95 cells despite the loss of AR expression (Figure 9F). These results document that AR is not suppressing the expression of NE or basal markers in the parental LN-95 ARSi-resistant ARPC cells.

### Transcriptome-based subtype clustering of PC PDXs.

**Figure 10:**
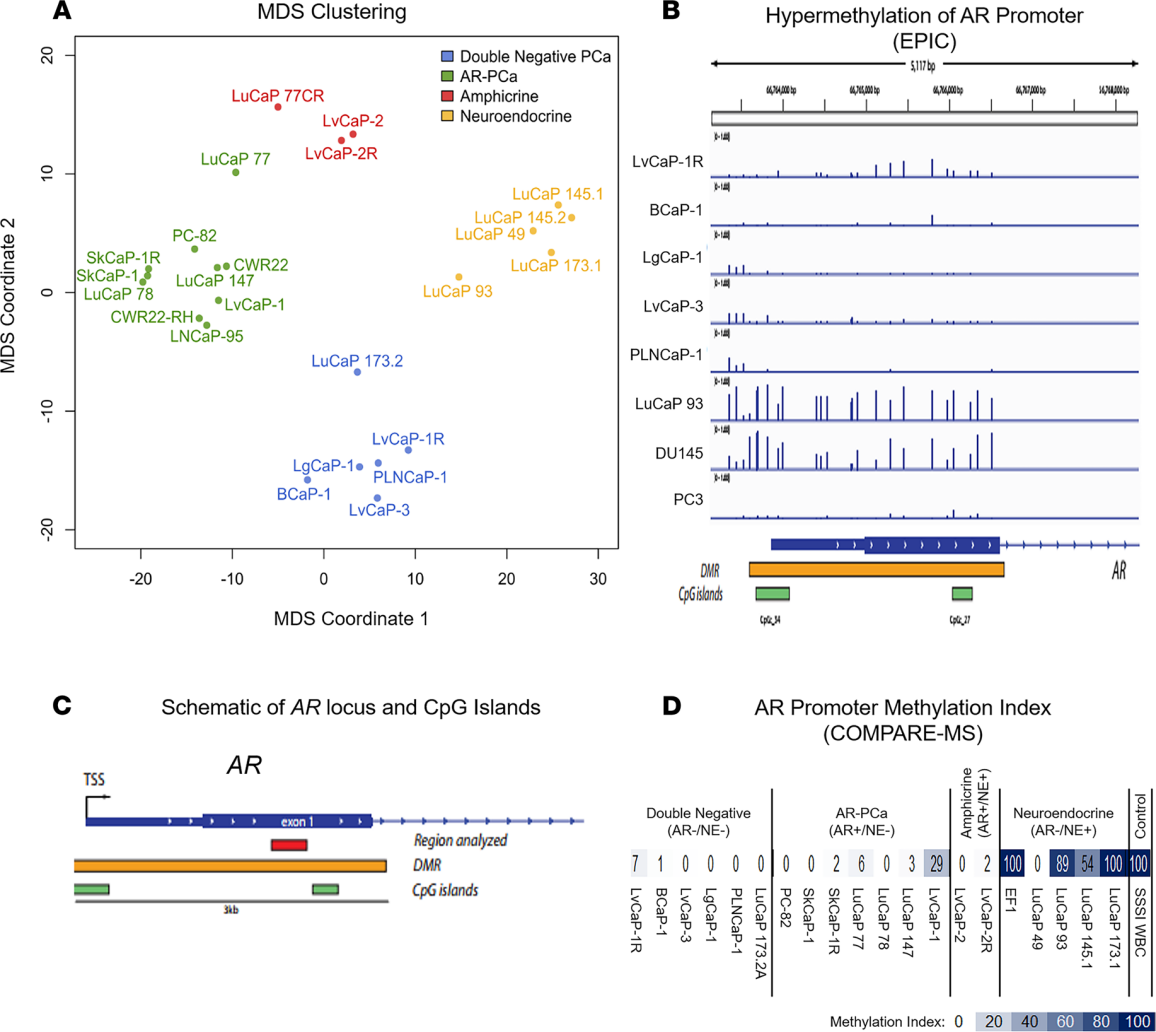
Clustering of lethal mCRPC PDXs and analysis of AR promoter methylation. (**A**) Clustering of PDX models based on multidimensional scaling. (**B**) Analysis of methylation levels at the single CpG level using Illumina EPIC arrays reveals hypermethylation of a region encompassing the transcriptional start site and the first exon of *AR* in LuCaP 93 and DU145 cells. (**C**) Schematic of the *AR* locus showing CpG islands, the putative differentially methylated region, and the region interrogated in this COMPARE-MS study. (**D**) Heat map of methylation indices in the first exon of AR in PDX lines as assessed by COMPARE-MS. mCRPC, metastatic castration-resistant prostate cancer; PDXs, patient-derived xenografts.

RNA-Seq analysis was performed to allow transcriptome-based clustering of an enlarged PDX series, including the newly developed DN PDXs described in this paper as well as previously characterized PC PDXs from both Johns Hopkins and the University of Washington Prostate Cancer Group (28, 29, 43). Four of these (i.e., PC-82, CWR22, SkCaP-1, LvCaP-1) are androgen-responsive ARPCs that regress when intact NSG immune-deficient adult male mice bearing these growing PDXs are castrated (29, 50). One (i.e., LvCaP-2) is an androgen-responsive AMPC, which regresses when tumor-bearing intact adult male mice are castrated, and 2 (i.e., LvCaP-2R and LuCaP77CR) are CR AMPC grown in castrated hosts (29, 43). The remaining 14 PDXs are also CR models grown in castrated hosts. Four (i.e., CWR22-RH, SkCaP-1R, 78CR, and 147CR) are CR ARPCs. Four (i.e., LuCaP 93, 145.1, 145.2, and 173.1) are NEPCs, and the remaining 6 (i.e., BCaP-1, LgCaP-1, LvCap-3, PLNCaP-1, LuCaP 173.2, and LvCaP-1R) are DNPCs. Multidimensional scaling (MDS) was performed based on the expression of a panel of 21 genes (10 gene “NE” and “AR” signatures plus AR; ref. 28). This MDS documents the expected clustering of ARPC versus NEPC PDXs (Figure 10A). The AMPC PDXs demonstrate an intermediate clustering due to the coexpression of AR and NE gene signatures (Figure 10A). In contrast, DNPC PDXs were distinctly clustered from the other phenotypes (Figure 10A).

### Androgen receptor promoter hypermethylation differed in DN versus NE PDXs.

DNPC and NEPC PDXs showed differential clustering despite neither one expressing AR. To determine whether these transcriptional differences were in part due to differences in AR transcriptional silencing, we performed genome scale and site-specific DNA methylation analyses. Infinium methylation EPIC array studies revealed a region of hypermethylation around the *AR* transcriptional start site that was present in the NEPC line LuCaP 93, but not in the DNPC lines LvCaP-1, BCaP-1, LgGaP-1, LvCaP-3, or PLNCaP-1 (Figure 10, B and C). As a positive control, AR^–^ DU145 PC cells were used because they are known to have hypermethylation around the transcriptional start site and expanding into the first exon (51, 52). As a negative control, PC3 cells were included (51, 52).

These findings were further corroborated by a targeted assessment of DNA methylation of the *AR* locus using COMPARE-MS (53). Of the 9 ARPCs evaluated, 8 showed minimal to no AR exon 1 DNA hypermethylation (Figure 10D), which is consistent with their high AR expression. Of note, in the 6 evaluated DNs lacking AR expression, no significant *AR* exon 1 DNA hypermethylation was detected (Figure 10, B and D). This is in contrast to the 6 NEPC PDXs evaluated, where 5 of the 6 lines showed DNA hypermethylation at this site (Figure 10, B and D), consistent with the lack of *AR* expression. These results suggest that the NEPC PDXs evaluated here were derived from cells that lost AR expression due to promoter hypermethylation whereas AR was transcriptionally silenced in DNPC PDXs via a different mechanism.

As a potential mechanism for the suppression of the NE phenotype within DN PDXs, a promoter methylation analysis of the 10 NE-related genes described in Figure 3B was performed. This analysis documented that only 4 of the 10 genes (i.e., *CHRNB2*, *PCSK1*, *ASCL1*, and *NKX2-1*) showed differential methylation consistent with the suppression of the NE phenotype in the DN versus NE PDXs (Supplemental Figure 4). Importantly, in only 1 of the genes (i.e., *CHRNB2*) was the promoter methylation pattern in the LvCaP-2/-2R amphicrine PDXs, consistent with that expected based on the NE phenotype in LuCaP 93 (Supplemental Figure 4).

## Discussion

Lineage plasticity of PC-initiating cells into a NE phenotype is supported by observations that multiple treatments can induce varying degrees of morphologic and phenotypic NE differentiation of prostate ARPC cells in vitro, including cAMP, IL-6, and serum starvation (54–57). In addition, ARS in ARPC cells can repress such NE differentiation in cell culture models (58–62). Based upon these results, it has been proposed that ARSi treatment of patients with mCRPC drives ARPCs into a more aggressive and lethal AR^–^ NEPC phenotype (28, 31–34). In particular, it has been suggested that such differentiation into NEPC requires the loss of ARS in combination with mutations in *PTEN, RB1*, and *TP53* together with the overexpression of DNMTs, EZH2, and/or SOX2 (28, 31–34, 63)

The present studies demonstrate that the lack of ARS in combination with these molecular alterations does not always necessitate progression of ARPCs to an ARSi-resistant NEPC phenotype. This conclusion is supported by previous reports. For example, Frigo and McDonnell demonstrated that only incomplete NE differentiation of ARPCs in vitro is produced by the inhibition of ARS with AR antagonists or siRNA-mediated downregulation of AR (62). Additionally, although HSP-90 inhibition downregulates AR expression, it has no effect on NE differentiation (62). In contrast, histone deacetylase (HDAC) inhibitor–induced epigenetic changes can promote such NE differentiation (62). Similarly, ADT is reported to activate the CREB/EZH2 axis, resulting in epigenetic activation of NE differentiation (64). Interestingly, the PDX models used in this study expressed both *CREB* and *EZH2*, and yet only a subset of the PDXs that lacked AR expression was NEPC. In addition, LNCaP cells stably transfected with *MYCN* phenotypically resemble NEPC with the upregulation of the NE markers and *EZH2* coupled with the downregulation of AR and androgen-regulated genes compared with parental cells (30). However, in the PDXs used in our studies, *MYCN* was highly expressed by BCaP-1, LVCaP-3, and LgCaP-1, and despite no AR expression, these cells did not exhibit NEPC characteristics. Hyperactive mTOR is reported to induce NE differentiation in vitro in LNCaP cells with the concurrent upregulation of IFN regulatory factor 1 and the downregulation of ARS associated with the upregulation of CDKN1A (also known as p21) and growth arrest (65). However, all of the AR^–^ PDXs reported herein expressed a high level of p21, but only a subset underwent NE differentiation and none were growth arrested. Thus, the present results document that ARSi resistance can occur without ARPC differentiation into a NEPC. These results suggest that in addition to the loss of AR, other molecular changes are needed for NEPC differentiation. There are several candidate changes for such NEPC drivers, including the downregulation of *REST* and *YAP*, in addition to the upregulation of *ASCL1*, a transcription factor important in neuronal development (15, 40, 66). Of note, it is significant that the DNPC PDXs did not downregulate *REST* and *YAP1* or upregulate *ASCL1* (Figure 3A); whereas, the NEPC PDXs did undergo these changes (Figure 3A).

Collectively, these results document that ARSi resistance occurs in both NEPC and DNPC via a loss of AR expression. In NEPC, this loss was frequently associated with hypermethylation and silencing of the AR promoter, which is consistent with previous studies supporting their derivation from ARPC cells via lineage plasticity (28, 31–34). In contrast, hypermethylation of the AR promoter was not detected in the current DNPC PDX series. In addition, differential promoter hypermethylation does not provide a simple explanation for the suppression of NE-related gene expression in the different phenotypes.

Importantly, all of the DNPC PDXs described herein had a mixture of malignant cells that heterogeneously expressed basal markers either alone or in combination with luminal markers. This phenotypic heterogeneity was present despite all cancer cells within each PDX having identical genetic driver mutations. There are at least 2 potential mechanisms to explain this phenomenon and the emergence of ARSi-resistant DNPC: (a) an initially AR^+^ cancer–initiating cell loses AR expression under ADT and acquires this phenotypic heterogeneity via lineage plasticity (i.e., adaptation); or (b) an AR^–^ prostate progenitor cell is the cancer-initiating cell and gives rise to malignant progeny heterogeneously expressing various combinations of basal and luminal markers (i.e., selection). In this second scenario, no promoter hypermethylation-dependent silencing of AR would be necessary for selective outgrowth of these DN cells under extreme androgen deprivation. Presently, these PDX models are being utilized to resolve whether adaption versus selection is the mechanism for the emergence of ARSi-resistant DNPC. Earlier studies demonstrated that PC with a luminal phenotype could be derived from genetically manipulated primary human benign prostate basal cells, suggesting that histology does not necessarily correlate with cell of origin (67).

Regardless of whether adaption or selection is responsible, approximately one-third of ARSi-resistant cancers are either DNPCs or NEPCs that lack AR expression, and the frequencies of such AR^–^ PCs are increasing (26). Thus, there is an urgent need for the development of therapies that do not depend upon AR activity for their efficacy (28, 68, 69). Thus, the PDXs characterized in the present report provide a credentialed platform for such drug development.

## Methods

Detailed procedures describing PDX establishment, cell culture, proliferation assays, cytogenetic, genetic and epigenetic characterization, plasmid construction and transfection of CRISPR/Cas9 vectors, isolation of clonal cell lines by FACS, RNA-Seq, DNA sequencing, methylation, Western blot, IHC, animal studies, and statistical analyses are included in the Supplemental Methods.

### Study approval.

Tissue collection for research was approved by the Johns Hopkins University School of Medicine IRB. Tumor specimens were acquired from patients with mCRPC who signed informed consent. All animal procedures were approved by the Johns Hopkins University School of Medicine Institutional Animal Care and Use Committee.

### Data availability.

The RNA-Seq data for this publication has been deposited in NCBI’s Gene Expression Omnibus and are accessible through accession number GSE160393 for the raw and mouse-gene subtracted PDX data, and GSE131985 for the LN-95 and AR-KO cells.

## Author contributions

WNB, YZ, and JTI conceived and designed the study. YZ, IMC, SLD, LA, RAP, BH, RC, AKM, and SLZ developed the methodology. YZ, IMC, SLD, LA, RAP, BH, RC, AKM, SLZ, JEH, and AMDM acquired the data (e.g., provided animals, acquired patients, provided facilities). WNB, YZ, IMC, SLD, LA, RAP, BH, RC, AKM, SLZ, JEH, JL, AMDM, EC, JX, SY, MCH, PSN, WGN, WBI, and JTI analyzed and interpreted the data (e.g., statistical analysis, bioinformatics, computational analysis). WNB, YZ, IMC, SLD, LA, RAP, BH, RC, AKM, SLZ, JEH, JL, AMDM, EC, JZ, SY, MCH, PSN, WGN, WBI, and JTI wrote, reviewed, and/or revised the manuscript. YZ, IMC, JEH, EC, and JL provided administrative, technical, or material support (e.g., reporting or organizing data, constructing databases, rapid autopsies, sharing resources). WNB and JTI supervised the study.

## Supplementary Material

Supplemental data

## Figures and Tables

**Table 1:**
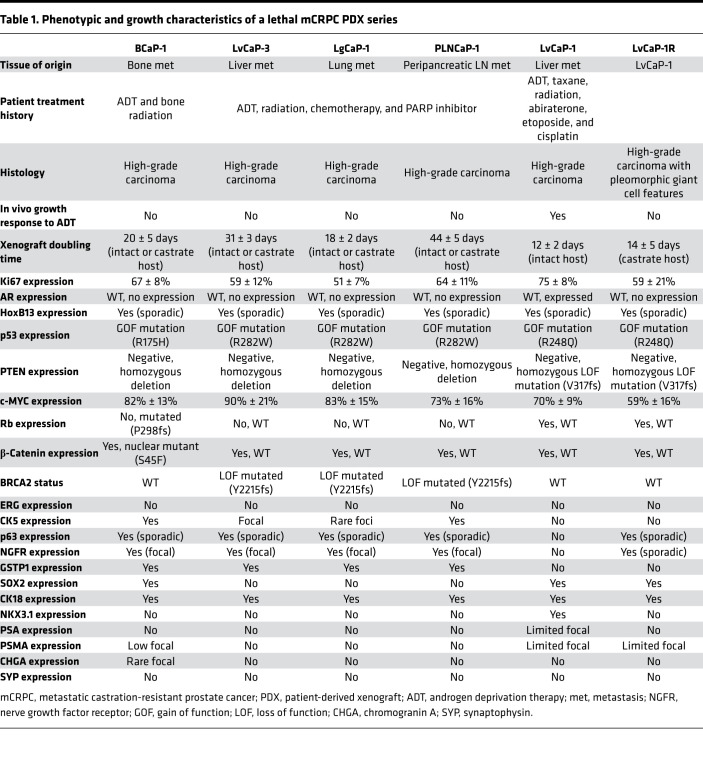
Phenotypic and growth characteristics of a lethal mCRPC PDX series

## Version 1 03/16/2021

In-Press Preview

## Version 2 04/22/2021

Electronic publication
