# Supplementary material for: Resistance to androgen receptor signaling inhibition does not necessitate development of neuroendocrine prostate cancer
Source: JCI Insight. 2021 Apr 22;6(8):e146827. doi: 10.1172/jci.insight.146827 (PMC8119192; doi:10.1172/jci.insight.146827)

Supplemental Materials and Methods

PDXs used in these studies

A series of 17 previously characterized prostate cancer PDXs established and maintained by the University of Washington Prostate Cancer Program (1, 2) and Johns Hopkins University Prostate Cancer Program (3) plus 6 additional novel PDXs established at Hopkins was utilized as described previously (3).

Novel PDX Establishment

Tumor specimens were acquired from mCRPC patients and were obtained from resection of distant metastases at rapid autopsy to limit warm ischemic time as much as possible (aiming for 4-8 hr after death). Harvested tumor tissues that were adjacent to the regions used for xenografting were evaluated by pathologists and tumor pieces were then prepared for implantation. NOD-SCID, triple immune-deficient NOG, NSG (NOD.Cg-PrkdcScidIl2rgtm1Wji/Szj), adult male mice obtained from the Sidney Kimmel Comprehensive Cancer Center (SKCCC) Animal Core Facility were used for tissue implantation. Tumor tissue was minced into small pieces, mixed at 40 with Matrigel obtained from BD Biosciences and then implanted at subcutaneous sites in the flank. At the time of initial grafting, mice were intact. Mice were monitored for up to 18 months post implantation for initial growth. Tumors that grew were serially passaged into intact male NSG mice. A PDX is considered established if there is active continuous growth for at least five serial passages. Once established, each PDXs was tested for its growth rate and androgen responsiveness. This was determined by inoculating the PDX into 10 intact male NSG mice and following the change in tumor volume over time using microcaliper measurement as described previously (4). When the cancers grew to approximately 200-400 mm3 half of the mice were castrated. The subsequent change in tumor volumes over time in the intact vs. castrated mice was used to define the androgen responsiveness of the PDX. If the PDX either regressed but then relapsed or grew at an equal rate in the intact vs. castrated host, it was subsequently serially passaged in castrated mice. Tumor samples were harvested from later passages (>3) for characterization. The human origin of each PDXs was confirmed by FISH analysis (**Supplemental Figure 5**) using human versus mouse specific centromere and telomere probes to identify human and mouse cell components as described previously (5). Genetic authentication for each of the PDXs was via short tandem repeat profiling conducted by the Johns Hopkins Genetic Resource Core Facility (**Supplemental Table 2**). When immune deficient mice are used as human tissue xenograft recipients, human B-cell lymphomas can develop from latent Epstein Barr Virus (EBV) infected B-cells that were initially present within the original human tissue are implanted (6). Each of the PDXs was negative by IHC staining for EBNA-2 protein characteristic of EBV-transformed cells documenting that none of the PDXs is a human B-cell lymphoma. Each PDX was also tested for their response to Enzalutamide (Enza). This was determined by growing the PDX in castrated mice to approximately 200-400 mm3 before randomizing the animals into a control group receiving daily oral gavage of 200 µl of vehicle only [i.e. 0.5 % carboxymethyl cellulose (Sigma Life Sciences)] vs. a group receiving daily oral gavage of 200 µl of vehicle containing Enza (MedChem Express, Monmouth Junction, NJ) to deliver 10 mg/kg/d or Abiraterone Acetate (Abi) to deliver 194

mg/kg/d. These doses have been documented to be effective in castrated mice against androgen sensitive ARPCs (3).

Serum PSA was measured as described previously (7).

Immunohistochemical (IHC) Staining

IHC staining was performed by the SKCCC Immunohistochemistry Core. Pretreatment conditions and primary antibody concentrations and source are summarized in (**Supplemental Table 3**). Images were taken using a Nikon DS-Qi1 Mc camera and NIS-Elements AR3.0 imaging software.

RNA Sequencing Analysis of the PDXs

RNA concentration, purity, and integrity was assessed by NanoDrop (Thermo Fisher Scientific, Inc.) and Agilent Tapestation. RNA-seq libraries were constructed from 1 ug total RNA using the Illumina TruSeq Stranded mRNA LT Sample Prep Kit according to the manufacturer's protocol. Barcoded libraries were pooled and sequenced on the Illumina HiSeq 2500 and Illumina Novaseq 6000 generating 50 bp paired end reads. Sequencing reads were mapped to the hg38 human and mm10 mouse genomes using STAR.v2.7.3a (8). Sequences aligning with higher specificity to the mouse genome deriving from potential contamination with mouse tissue were removed from the analysis using XenofiltR. Gene level abundance was quantitated from the filtered human alignments in R using the GenomicAlignments Bioconductor package (9) and normalized to kilobase of transcript per million mapped reads (FPKM) using edgeR (10). The expression values are shown as log2 FPKM. Sample phenotypic groups were assigned using classical multidimensional scaling (MDS) calculated with the cmdscale function in R on the expression profile of 21 genes from the combined lists of the 10-gene "NE" and "AR" signatures in (2) plus AR. The distance metric was "euclidean" calculated by the dist function on the columns (samples). The LvCaP-1, LN-95, CWR22, and PC-82 PDXs, which are AR-positive adenocarcinomas derived from hormonally-untreated primary prostate cancer patients, were used as additional canonical AR-PCa examples for comparison. These PDXs were obtained and propagated as previously described (11). LuCaP 49, 93, 145.1, and 145.2 were generated as previously described and used as representative examples of NEPC PDXs (12).

DNA Sequencing of the PDXs

A customized NGS panel targeting 355 cancer related genes was used to sequence DNA samples. The probes for capturing exon regions in these genes were manufactured by Roche NimbleGen. SeqCap EZ Library SR User's Guide (Roche, Pleasanton, CA) was followed for library preparation and capture of targeted sequences. Paired-end sequencing of 2 x 150 bp was performed on an Illumina NextSeq500. The median coverage for these 64 samples was at 324x. Paired-end reads was aligned to the GRCh37 version of the human genome using Burrows-Wheeler Aligner v0.7 to generate BAM files (13). After sorting the BAM files using samtools, PCR duplicates marked using Picard and realignment around putative gaps was performed using the Genome Analysis Toolkit (GATK) v3.2-2. Variant calling was performed with the GATK Haplotype caller. ANNOVAR (http://annovar.openbioinformatics.org/en/latest) and snpEff were used for annotating variants and for retrieving information on variants in the population-based studies such as the 1000 Genomes Project (www.1000genomes.org), NHLBI-ESP 6500 exomes, or ExAC (http://exac.broadinstitute.org/) or

gnomAD (http:// http://gnomad.broadinstitute.org/), and clinical databases such as the Human Gene Mutation Database (HGMD) (14) and ClinVa (15). Pathogenicity of variants is defined based on American College of Medical Genetics and Genomics (ACMG) criteria (16). Specifically, pathogenic and likely pathogenic mutations are defined as 1) all protein truncating mutations unless their allele frequency is 5% or higher in any racial group in population databases or is reported as benign or likely benign in the ClinVar, and 2) nonsynonymous changes if their allele frequency is less than 5% and reported as pathogenic and likely pathogenic mutations in the ClinVar.

AR methylation analysis

For *AR* locus-specific DNA methylation analyses, a previously validated assay combining methylated-DNA precipitation and methylation-sensitive restriction enzyme digestion (COMPARE-MS) was used (17). In brief, DNA samples were digested with AluI and HhaI (New England Biolabs) and methylated DNA fragments were enrichment using recombinant MBD2-MBD (Clontech) immobilized on magnetic Tylon beads (Clontech). The precipitated DNA containing methylated DNA fragments was eluted and subjected to quantitative real-time PCR using IQ SYBR Green Supermix (Biorad) with primers specific to the CpG island in the first exon of AR F: 5'-CATGTACGCCCCACTTTTG; R: 5'-CTCTCGCCTTCTAGCCCTTT. For quantitative assessment of locus specific methylation levels, ct-values of the samples of interest were normalized to ct-values of the positive control (in vitro fully methylated male genomic DNA) and calculated methylation indices (ranging from 0.0 to1.0) were used to derive methylation heatmaps. In addition, genome scale methylation analyses of the prostate cancer cell lines PC3 and DU145 as well as LuCaP 93, LvCaP-1, BCaP-1, LgCaP-1, LvCaP-3 and PLNCaP-1 PDXs were carried out using Infinium Methylation EPIC BeadChip arrays (Illumina) as described previously (18). Raw data were analyzed using the Minfi Bioconductor package and visualized using the IGV (19, 20).

Cell Culture

LN-95 cells is an androgen-independent cell line derived from parental LNCaP cells as previously reported (21). LN-95 cells were cultured in phenol red free RPMI 1640 medium (Invitrogen, Carlsbad, CA) supplemented with 10% charcoal-stripped FBS (CSS, Invitrogen, Carlsbad, CA). All the AR isoforms knockout clones were cultured in RPMI-1640 Medium (Invitrogen, Carlsbad, CA) with 10% fetal bovine serum (FBS, Sigmal-Aldrich, St. Louis, MO). All cell lines were maintained at 37 °C in 5% CO2. Cell growth was as described previously (22).

Plasmid Construction and Transfection

Generation of LN-95 total AR KO cell lines was described previously (3). Briefly, the CRISPR-Cas9 vector used for AR gene editing was pSpCas9(BB)-2A-GFP (Addgene plasmid ID: 48138). The guide RNAs were designed using CRISPR guide design tool: http://crispr/mit.edu/. Guide RNAs targeting different AR exons were listed below: sgAR-Exon1: CCGCCGTCCAAGACCTACCG. To construct the expression plasmids for guide RNAs, all guide RNA oligo pairs were annealed and cloned into the BbsI restriction site of pSpCas9(BB)-2A-GFP plasmid, and the correct plasmids were verified by sequencing from the U6 promoter using the U6-Fwd primer (GAGGGCCTATTTCCCATGATTCC). One day before transfection, 400,000 LN-95 cells were seeded in each well of 6-well plates in phenol-red free RPMI1640 supplemented with 10% charcoal-stripped FBS and

1% Penicillin/Streptomycin to achieve 60% confluence. The next day, each well was transfected with 2.5 μg of plasmid expressing the desired AR-guide RNA or empty GFP vector control with 3.75 μL of Lipofectamine 3000 (ThermoFisher, cat # L3000008).

Isolation of Clonal Cell Lines by FACS

After 48 hours of transfection, the cells were dissociated by TrypLE and resuspended into the FACS sorting buffer (PBS + 2% BSA + 20 mM EDTA). The cells were filtered into cell strainer tube, and sorted by Flow Cytometer (S3e Cell Sorter, BioRad) for GFP positive population. The sorted cells were then seeded in 100 mm dishes in phenol-red free RPMI1640 supplemented with 10% charcoal-stripped FBS and 1% Penicillin/Streptomycin, with 600 cells/dish and allowed to grow into colonies. Approximately 4 weeks later, 10-20 colonies were picked from each transfected cells using cloning discs (diam. 3.2 mm, #Z374431, Sigma-Aldrich) and transferred into 24 well plates to expand for further analyses.

Genotyping of Clonal Cell Lines

Once colonies were expanded into each well of 6-well plates, genomic DNA was isolated using QiaAmp DNA mini kit (cat #51306, Qiagen) and amplified using GoTaq DNA Polymerase (M300, Promega) with verification primer sets as listed below: AR-E1-fwd: GACTACCGCATCA-TCACAGC. AR-E1-rev: AGGTTGCTGTTCCTCATCCA. Two colonies from cells transfected with AR-E1-sgRNA were confirmed carrying homozygous insertion (+1) and deletion (Δ2) respectively. The on-target editing of AR genomic regions were verified by Sanger sequencing of the PCR products. The indel mutations of AR gene generated by on-target editing all resulted in premature stop codon as predicted by ExPASy Translate Tool (http://web.expasy.org/translate/).

AR Isoforms and HOXB13 Protein Analysis

The whole cell lysates were prepared using RIPA buffer (Pierce) according to the manufacture's suggestions. Nuclear and cytosolic lysates were extracted by Nuclear and Cytoplasmic Extraction Reagents (Pierce). Protein samples concentration was quantified using BCA Protein Assay Kit (Pierce) and equal amount of protein samples were resolved on 4%-12% gradient SDA-PAGE gels and subjected to western blotting with anti-AR(441) (Santa Cruiz Biotechnology), anti-AR-V7 (Precision, AG10008) and anti-AR-CTD (Sigma, SP242 SAB5500007). For HOXB13 western blotting, whole cell lysates were resolved and probed with appropriate antibody (R&D, AF8156).

Cellular Quantitation HOXB13 in Normal Prostate

Formalin-fixed, paraffin embedded sections of human normal prostate tissue from a 17 year old organ donor was dual fluorescent immune histochemical (IHC) labeled for HoxB13 and chromograrin A (CGA) antibodies [anti-HoxB13 (1:500 dilution; catalog# ab201682, AbCam) and anti-CGA (1:500; catalog# MA5-14536, Thermofisher Scientific] using OPAL 4-color IHC kit (catalog # NEL810001KT, Akoya Biosciences) following the manufacturer's instructions. Antibodies for the first round were stripped off in Antigen Unmasking Buffer by microwave and followed by the second round of staining. Slides were counterstained with DAPI and mounted with Prolong Gold (catalog# P10144, ThermoScientific).

IHC slides were imaged using the TissueFAXS Plus (Tissue Gnostics, Vienna, Austria) automated microscopy workstation equipped with a Zeiss Z2 Axio Imager microscope (Carl Zeiss Microscopy, LCC, Thornwood, NY, U.S.A.) and appropriate fluorescence filter sets. Exposure times for each channel (HOXB13, chromogranin and DAPI) were optimized to minimize pixel saturation while maximizing signal to noise for each fluorescent marker and the entire tissue section was then scanned with a 40X objective in an automated fashion. The digitized IF signals were then assessed using the TissueQuest 6.0 software (Tissue Gnostics) module to analyze the fluorescent images with precise nuclear segmentation on DAPI. The HOXB13 fluorescence staining intensity was captured for 97 individual cells with clear and robust cytoplasmic staining for CGA with an associated nucleus. HOXB13 fluorescence was also determined for 97 nearby basal ductal epithelial cells judged by location relative to stroma and relative to adjacent luminal epithelial cells, and judged also by nuclear size and shape in keeping with basal epithelial cell phenotype(e.g. smaller and more compact that the adjacent luminal layer, and also not possessing spindle or cylindrically shaped nuclei; as are typically seen for stromal smooth muscles and fibroblasts) and for 24 corresponding luminal epithelial cells which displayed markedly higher levels of HOXB13 in all cases. For each cell type, the ratio of total nuclear HOXB13 staining intensity to the nuclear area (DAPI staining) was determined, in order to further correct for fractional nuclei in the cut tissue section.

Immunohistochemistry (IHC) for LN-95 Cell Lines

Cell pellets were fixed in 10% neutral-buffered formalin and embedded in paraffin. Sections were cut and processed for IHC. For AR-V7 IHC (RM7, RevMAb Biosciences, San Francisco, CA), slides were first deparaffinized, followed by antigen retrieval (Antigen Unmasking Solution, Tris-Based High pH, H-3301, Vector Lab , Burlingame, CA, USA) at 95℃for 20 min. The slides were incubated with the anti-human AR-V7 antibody (1:200, RevMAb Biosciences, 31-1109-00) for 70 min at room temperature. The secondary antibody anti-rabbit poly HRP IgG (PowerVision, PV6118) was incubated with the slides for 30 min at room temperature. For AR N-terminus IHC, de-paraffinized slides were incubated in Antigen Unmasking Solution (Tris-Based High pH, H-3301, Vector Lab, Burlingame, CA, USA) at 95℃ for 40 min. The slides were incubated with anti-AR-441 (1:200, Santa Cruiz Biotechnology) for 1h at room temperature. The slides were then incubated with anti-mouse poly HRP IgG (PV6113) for 30min RT. All slides were then visualized by DAB staining and counterstained with 50% Haematoxylin for 2 min. Representative images were taken by Olympus 40X Bright Field microscope with digital camera.

RNA Sequencing of In Vitro Cell Lines

Parental LN-95, and AR-KO cells were subjected to RNA-Seq following the standard Trizol/RNeasy total RNA Prep Kit. The quality of the RNA extracted were verified by Qubit 3 (Invitrogen) and Bioanalyzer (Agilent) and sequenced using the Illumina HiSeq 2000 platform (Illumina Inc, San Diego, CA). An average of ~100 million reads per sample were generated. Sequences were aligned to UCSC hg19 genome building using TopHat, and the insertion or deletions were visualized using Integrative Genomics Viewer (IGV) (19). Read counts were obtained using HTSeq, and normalized per kilo base-pair gene length and per million reads library size (RPKM).

Statistical Analysis

Results are representative and expressed as mean +/- SEM. A p-value of <0.05, determined by Student's T-test or ANOVA test when appropriate, was considered statistically significant.

Study Approval

Tissue collection for research was approved by the Johns Hopkins University School of Medicine IRB. Tumor specimens were acquired from mCRPC patients who signed informed consent. All animal procedures were approved by the Johns Hopkins University School of Medicine Institutional Animal Care and Use Committee.

Data Availability

The RNA-seq data for this publication has been deposited in NCBI's Gene Expression Omnibus and are accessible through accession number GSE160393 for the raw and mouse-gene subtracted PDX data, and GSE131985 for the LN-95 and AR-KO cells.

Supplemental References

1. Nguyen HM, Vessella RL, Morrissey C, Brown LG, Coleman IM, Higano CS, Mostaghel EA, Zhang X, True LD, Lam HM, et al. LuCaP Prostate Cancer Patient-Derived Xenografts Reflect the Molecular Heterogeneity of Advanced Disease an--d Serve as Models for Evaluating Cancer Therapeutics. *Prostate.* 2017;77(6):654-71.
2. Bluemn EG, Coleman IM, Lucas JM, Coleman RT, Hernandez-Lopez S, Tharakan R, Bianchi-Frias D, Dumpit RF, Kaipainen A, Corella AN, et al. Androgen Receptor Pathway-Independent Prostate Cancer Is Sustained through FGF Signaling. *Cancer Cell.* 2017;32(4):474-89 e6.
3. Zhu Y, Dalrymple SL, Coleman I, Zheng SL, Xu J, Hooper JE, Antonarakis ES, De Marzo AM, Meeker AK, Nelson PS, et al. Role of androgen receptor splice variant-7 (AR-V7) in prostate cancer resistance to 2nd-generation androgen receptor signaling inhibitors. *Oncogene.* 2020;39(45):6935-49.
4. Sedelaar JP, and Isaacs JT. Tissue culture media supplemented with 10% fetal calf serum contains a castrate level of testosterone. *Prostate.* 2009;69(16):1724-9.
5. Vander Griend DJ, Konishi Y, De Marzo AM, Isaacs JT, and Meeker AK. Dual-label centromere and telomere FISH identifies human, rat, and mouse cell contribution to Multispecies recombinant urogenital sinus xenografts. *Prostate.* 2009;69(14):1557-64.
6. Brennen WN, and Isaacs JT. The what, when, and why of human prostate cancer xenografts. *Prostate.* 2018;78(9):646-54.
7. Michiel Sedelaar JP, Dalrymple SS, and Isaacs JT. Of mice and men--warning: intact versus castrated adult male mice as xenograft hosts are equivalent to hypogonadal versus abiraterone treated aging human males, respectively. *Prostate.* 2013;73(12):1316-25.
8. Dobin A, Davis CA, Schlesinger F, Drenkow J, Zaleski C, Jha S, Batut P, Chaisson M, and Gingeras TR. STAR: ultrafast universal RNA-seq aligner. *Bioinformatics.* 2013;29(1):15-21.
9. Lawrence M, Huber W, Pages H, Aboyoun P, Carlson M, Gentleman R, Morgan MT, and Carey VJ. Software for computing and annotating genomic ranges. *PLoS Comput Biol.* 2013;9(8):e1003118.
10. Robinson MD, McCarthy DJ, and Smyth GK. edgeR: a Bioconductor package for differential expression analysis of digital gene expression data. *Bioinformatics.* 2010;26(1):139-40.
11. Isaacs JT, D'Antonio JM, Chen S, Antony L, Dalrymple SP, Ndikuyeze GH, Luo J, and Denmeade SR. Adaptive auto-regulation of androgen receptor provides a paradigm shifting rationale for bipolar androgen therapy (BAT) for castrate resistant human prostate cancer. *Prostate.* 2012;72(14):1491-505.
12. Navone NM, van Weerden WM, Vessella RL, Williams ED, Wang Y, Isaacs JT, Nguyen HM, Culig Z, van der Pluijm G, Rentsch CA, et al. Movember GAP1 PDX project: An international collection of serially transplantable prostate cancer patient-derived xenograft (PDX) models. *Prostate.* 2018;78(16):1262-82.
13. Li H, and Durbin R. Fast and accurate short read alignment with Burrows-Wheeler transform. *Bioinformatics.* 2009;25(14):1754-60.

14. Stenson PD, Ball EV, Mort M, Phillips AD, Shiel JA, Thomas NS, Abeysinghe S, Krawczak M, and Cooper DN. Human Gene Mutation Database (HGMD): 2003 update. *Hum Mutat.* 2003;21(6):577-81.
15. Landrum MJ, Lee JM, Riley GR, Jang W, Rubinstein WS, Church DM, and Maglott DR. ClinVar: public archive of relationships among sequence variation and human phenotype. *Nucleic Acids Res.* 2014;42(Database issue):D980-5.
16. Green RC, Berg JS, Grody WW, Kalia SS, Korf BR, Martin CL, McGuire AL, Nussbaum RL, O'Daniel JM, Ormond KE, et al. ACMG recommendations for reporting of incidental findings in clinical exome and genome sequencing. *Genet Med.* 2013;15(7):565-74.
17. Yegnasubramanian S, Lin X, Haffner MC, DeMarzo AM, and Nelson WG. Combination of methylated-DNA precipitation and methylation-sensitive restriction enzymes (COMPARE-MS) for the rapid, sensitive and quantitative detection of DNA methylation. *Nucleic Acids Res.* 2006;34(3):e19.
18. Pidsley R, Zotenko E, Peters TJ, Lawrence MG, Risbridger GP, Molloy P, Van Djik S, Muhlhausler B, Stirzaker C, and Clark SJ. Critical evaluation of the Illumina MethylationEPIC BeadChip microarray for whole-genome DNA methylation profiling. *Genome Biol.* 2016;17(1):208.
19. Robinson JT, Thorvaldsdottir H, Winckler W, Guttman M, Lander ES, Getz G, and Mesirov JP. Integrative genomics viewer. *Nat Biotechnol.* 2011;29(1):24-6.
20. Aryee MJ, Jaffe AE, Corrada-Bravo H, Ladd-Acosta C, Feinberg AP, Hansen KD, and Irizarry RA. Minfi: a flexible and comprehensive Bioconductor package for the analysis of Infinium DNA methylation microarrays. *Bioinformatics.* 2014;30(10):1363-9.
21. Pflug BR, Reiter RE, and Nelson JB. Caveolin expression is decreased following androgen deprivation in human prostate cancer cell lines. *Prostate.* 1999;40(4):269-73.
22. Litvinov IV, Vander Griend DJ, Xu Y, Antony L, Dalrymple SL, and Isaacs JT. Low-calcium serum-free defined medium selects for growth of normal prostatic epithelial stem cells. *Cancer Res.* 2006;66(17):8598-607.

Supplemental Table and Figure Legends

Supplemental Table 1: Characteristics of patient-derived samples, including metastatic lesions from which the PDXs were derived, based on immunohistochemical staining.

Supplemental Table 2: STR profile of the novel PDXs and LN-95 xenografts.

Supplemental Table 3: IHC pre-treatment and primary antibody details.

Supplemental Figure 1: **Expression of prostate-specific differentiation markers. a.)** *in vivo* serum PSA of DN-PDXs (i.e. BCap-1 , LvCaP-3, LgCaP-1, PLNCaP-1, and LvCaP-1R) vs. ARPCs (i.e. LvCap-1, CWR22, PC-82) or AMPCs (i.e. LvCaP-2 and LvCap-2R) (n = 3-28). **b.)** Western blot for HOXB13 documenting prostatic origin of each of the DN PDX. ARPC LvCaP-1 and the AMPC LvCaP-2 are used as positive controls. **c.)** *in vitro* PSA secretion from LNCaP cultured in standard and CS-media conditions, in addition to LN-95 and the AR-KO derivative in CS-media (n = 3-8). Box plots display the median and interquartile range (whiskers = min/max values).

Supplemental Figure 2: Treatment histories of patients from which each of the novel PDX model was derived.

Supplemental Figure 3: ***in vitro* morphology of LNCaP, LN-95, and LN-95 total AR-KO #1 and AR-KO #2.** Cells grown in media indicated in parentheses. 400x.

Supplemental Figure 4: Lack of differential promoter methylation of NE-related genes across PDX phenotypes. Analysis of methylation levels at the single CpG level using Illumina EPIC arrays reveals inconsistent differential hypermethylation of a region encompassing the transcriptional start site of **a.)** *CHRNB2*, **b.)** *PCSK1*, **c.)** *ASCL1*, **d.)** *NKX2-1*, **e.)** *CHGA*, **f.)** *CHGB*, and **g.)** *SYP*. Yellow box highlights differential promoter methylation in *CHRNB2*, *PCSK1*, *ASCL1*, and *NKX2-1*, but not in *CHGA*, *CHGB*, and *SYP*.

Supplemental Figure 5: **Centromere-telomere PNA FISH (Cen/Tel PNA FISH) on BCaP-1 PDX tissue showing human cells surrounding rodent endothelial cells.** A human- and mouse-specific FITC-labeled centromeric FISH probe (green) was combined with a Cy3-labeled telomere FISH probe (red), which highlights the longer rodent telomeres counterstained with DAPI (blue) to identify nuclei. Such a method allows unambiguous discernment between human cells (Cen-positive; Tel-dim) denoted by arrows and mouse cells (Cen-positive; Tel-bright) denoted by triangles.

Supplemental Tables and Figures

Supplemental Table 1

Autopsy	A001								A009					A0011						
**Site**	Prostate (P1) (RP)	Liver (M5)	Perigastric LN (M38)	Lung (M40)	Hilar LN (M45)	Sub-hepatic LN	Diaphragm (M57)	Bone (M63)	Bone – L4	Bone – R. Tibia	Prostate (no RP)	Subcarinal LN	R. Central Liver	Liver	Pericardial LN	Peri-pancreatic LN	Lung – R. Lower	Lung – Left	Diaphragm	Adrenal – Left
**PDX**		**LvCaP-1/ -1R**								**BCaP-1**				**LvCaP-3**		**PLNCaP-1**	**LgCaP-1**			
**Prostate**																				
PSA									~90% Strong Positive	Negative	100% Strong Pos	Negative	Negative	Negative	Negative	Negative	Negative	Negative	Negative	Negative
PSAP									~80% Strong Pos	Negative	100% Strong Pos	Negative	Negative							
PSMA									~100% Strong Pos	40% Strong Pos	100% Strong Pos	30% Strong	Negative							
NKX3.1	Positive	Positive	Positive	Positive	Positive	Positive	Positive	Positive	~100% Strong Pos	Negative	80% Strong Pos	Negative	Negative							
AR	Positive	Strong Positive	Strong Positive	Strong Positive	Strong Positive	Strong Positive	Strong Positive	Strong Positive	~100% Strong Pos	Negative	80% Strong Pos	Negative	Negative	Negative	Negative	Negative	Negative	Negative	Negative	Negative
HOXB13									Mostly Strong Pos	Focal Weak Pos	Mostly Strong Pos	Focal Weak Pos	Negative							
P501S/ Prostein														5-10%	60-80%	80-90%	80%	60%	100%	70%
**Proliferation**																				
KI67		>25%	>25%	>25%	>25%	>25%	>25%	>25%	40%	90%	20%	90%	90%	80%	80-90%	70-90%	80-90%	80%	80-90%	90%
**Oncogene**																				
ERG	Negative	Negative	Negative	Negative	Negative	Negative	Negative	Negative	Negative	Negative	Negative	Negative	Negative	Negative	Negative	Negative	Negative	Negative	Negative	Negative
**Tumor Suppressor**																				
PTEN	Negative (100%)	Negative (100%)	Negative (100%)	Negative (100%)	Negative (100%)	Negative (100%)	Negative (100%)	Negative (100%)	Negative (100%)	Negative (100%)	Negative (100%)	Negative (100%)	Negative (100%)	Negative (100%)	Negative (100%)	Negative (100%)	Negative (100%)	Negative (100%)	Negative (100%)	Negative (100%)
RB1	Positive	Positive	Positive	Positive	Positive	Positive	Positive	Positive	Negative (100%)	Negative (100%)	Negative (100%)	Negative (100%)	Negative (100%)	Negative (100%)	Negative (100%)	Negative (100%)	Negative (100%)	Negative (100%)	Negative (100%)	Negative (100%)
P53 (mutated)	Positive	Positive	Positive	Positive	Positive	Positive	Positive	Positive	Positive	Positive	Positive	Positive		Positive	Positive	Positive	Positive	Positive	Positive	Positive
**Epithelial**																				
CK8									~80% Strong Pos	100% Strong Pos	100% Strong Pos	100% Strong	100% Strong Pos							
CK18									Focally Strong Pos	80% Strong Pos	70% Strong Pos	40% Strong	80% Strong Pos							
**Neuro-endocrine**																				
CHG									Negative	Negative	Negative	Negative	Negative	Negative	Negative	Negative		Negative	Negative	Negative
SYP									<5% Moderate Pos	Negative	Negative	Negative		5% Positive	Negative	Negative		Negative	Negative	Negative
FOXA2									~10% Moderate Pos	100% Strong Pos	100% Strong Pos	100% Strong	100% Strong Pos							

Supplemental Table 2

PDX	AMEL	CSF1PO	D13S317	D16S539	D21S11	D5S818	D7S820	TH01	TPOX	vWA
**LN-95**	X	10, 11, 12, 13	10, 12, 13, 14	10, 11	29, 33.2, 34.2	10, 11, 12	9, 9.1, 9.2, 9.3, 10.3	9	9, 10	16, 17, 18, 19
**LVCaP-1**	X, Y	10, 11	10	12, 13	28, 30	10	11, 12	7, 9	8, 12	17
**LvCaP-1R**	X, Y	10, 11	10	13	28, 29, 30	10	11, 12	7, 9	8, 12	17
**LvCaP-2**	X, Y	12	13	21	28	12	12	6, 9.3	8	16, 18
**LvCaP-2R**	X, Y	10, 12	13	12	28	12	12	6, 9.3	8	16, 18
**BCaP-1**	X, Y	10, 11	12	9, 12	28	9, 12	9, 12	9	8, 11	18, 20
**LvCaP-3**	X, Y	12	12	12	30, 31.2	11	8	7, 9	8	19
**LgCaP-1**	X, Y	12	12	12	30, 31.2	11	8	7, 9	8	19
**PLNCaP-1**	X, Y	12	12	12	30, 31.2	11	8	7	8	19

Supplemental Table 3

Antigen	Catalog #	Vendor	Staining	Ab Dilution	Retrieval	Detection	Amplification	Chromogen	Counter-stain
**AE1/3**	ab961	AbCam	Ventana Discovery Ultra	1:10	On board, CC1	HQ anti-mouse		DAB	Hematoxylin
**AR**	sc-816	Santa Cruz Biotechnologie s	Ventana Discovery Ultra	1:200	On board, CC1	HQ anti-rabbit		DAB	Hematoxylin
**β-catenin (active) (non-phospho Ser33/37/Thr41)**	8814	Cell Signaling Technologies	Manual	1:600	Steamed in Citrate pH 6.0 (Vector Labs, H-3300)	Powervision anti-rabbit HRP (Leica, PV6119)		DAB	Hematoxylin
**β-catenin (total)**	ab32572	AbCam	Manual	1:200	Steamed in Citrate pH 6.0 (Vector Labs, H-3300)	Powervision anti-rabbit HRP (Leica, PV6119)		DAB	Hematoxylin
**CD68**	Ab125212	AbCam	Manual	1:250	Steamed in Target Retrieval Solution (Dako, S1699)	Powervision anti-rabbit HRP (Leica, PV6119)		DAB	Hematoxylin
**Chromogranin A**	MA5-14536	Thermo Scientific	Ventana Discovery Ultra	1:500	On board, CC1	HQ anti-rabbit		DAB	Hematoxylin
**CK14**	ab51054	AbCam	Manual	1:50	Steamed in Target Retrieval Solution (Dako, S1699)	Powervision anti-rabbit HRP (Leica, PV6119)		DAB	Hematoxylin
**CK18**	MAB3236	Millipore	Manual	1:100	Steamed in Target Retrieval Solution (Dako, S1699)	Powervision anti-mouse HRP (Leica, PV6114)		DAB	Hematoxylin
**CK5**	905501	BioLegend	Ventana Discovery Ultra	1:5000	On board, CC1	HQ anti-rabbit		DAB	Hematoxylin
**CK8**	ab53280	AbCam	Manual	1:1800	Steamed in Target Retrieval Solution (Dako, S1699)	Powervision anti-rabbit HRP (Leica, PV6119)		DAB	Hematoxylin
**c-MYC**	1472-1	Epitomics	Ventana Discovery Ultra	1:200	On board, CC1	HQ anti-rabbit	HQ Amp	DAB	Hematoxylin
**EBNA-2**	ab90543	AbCam	Manual	1:250	Steamed in Citrate pH 6.0	Powervision anti-mouse HRP (Leica, PV6114)		DAB	Hematoxylin
**GSTP1**	m00394-2	Boster Bio	Ventana Discovery Ultra	1:2000	On board, CC1	HQ anti-rabbit		DAB	Hematoxylin
**HOXB13**	ab201682	AbCam	Ventana Discovery Ultra	1:1000	On board, CC1	HQ anti-rabbit		DAB	Hematoxylin
**Ki67**	ab16667	AbCam	Ventana Discovery Ultra	1:200	On board, CC1	HQ anti-rabbit		DAB	Hematoxylin
**NGFR**	ab42987	AbCam	Ventana Discovery Ultra	1:250	On board, CC1	HQ anti-rabbit		DAB	Hematoxylin
**NKX3.1**	314	Athena	Ventana Discovery Ultra	1:750	On board, CC1	HQ anti-rabbit		DAB	Hematoxylin
**p53 (D07)**	790-2912	Roche Diagnostics	Ventana Discovery Ultra	RTU	On board, CC1	OptiView (rabbit + mouse)	OV Amp	DAB	Hematoxylin
**p63**	13109S	Cell Signaling Technologies	Ventana Discovery Ultra	1:200	On board, CC1	HQ anti-rabbit	HQ Amp	DAB	Hematoxylin
**PSA**	m0750	Dako	Manual	1:25	Steamed in Target Retrieval Solution (Dako, S1699)	Powervision anti-mouse HRP (Leica, PV6114)		DAB	Hematoxylin
**PSMA**	1215S	Cell Signaling Technologies	Manual	1:100	Steamed in Target Retrieval Solution (Dako, S1699)	Powervision anti-rabbit HRP (Leica, PV6119)		DAB	Hematoxylin
**PTEN**	9188S	Cell Signaling Technologies	Ventana Discovery Ultra	1:100	On board, CC1	HQ anti-rabbit		DAB	Hematoxylin
**Rb**	ab181616	AbCam	Ventana Discovery Ultra	1:400	On board, CC1	HQ anti-rabbit	HQ Amp	DAB	Hematoxylin
**SMA**	m0851	Dako	Ventana Discovery Ultra	1:1000	On board, CC1	HQ anti-mouse		Discovery Purple	Hematoxylin
**Sox2**	3579	Cell Signaling Technologies	Manual	1:100	Steamed in Target Retrieval Solution (Dako, S1699)	Powervision anti-rabbit HRP (Leica, PV6119)		DAB	Hematoxylin
**SYP**	790-4407	Roche	Ventana Discovery Ultra	RTU	On board, CC1	HQ anti-rabbit		DAB	Hematoxylin

Supplemental Figure S1

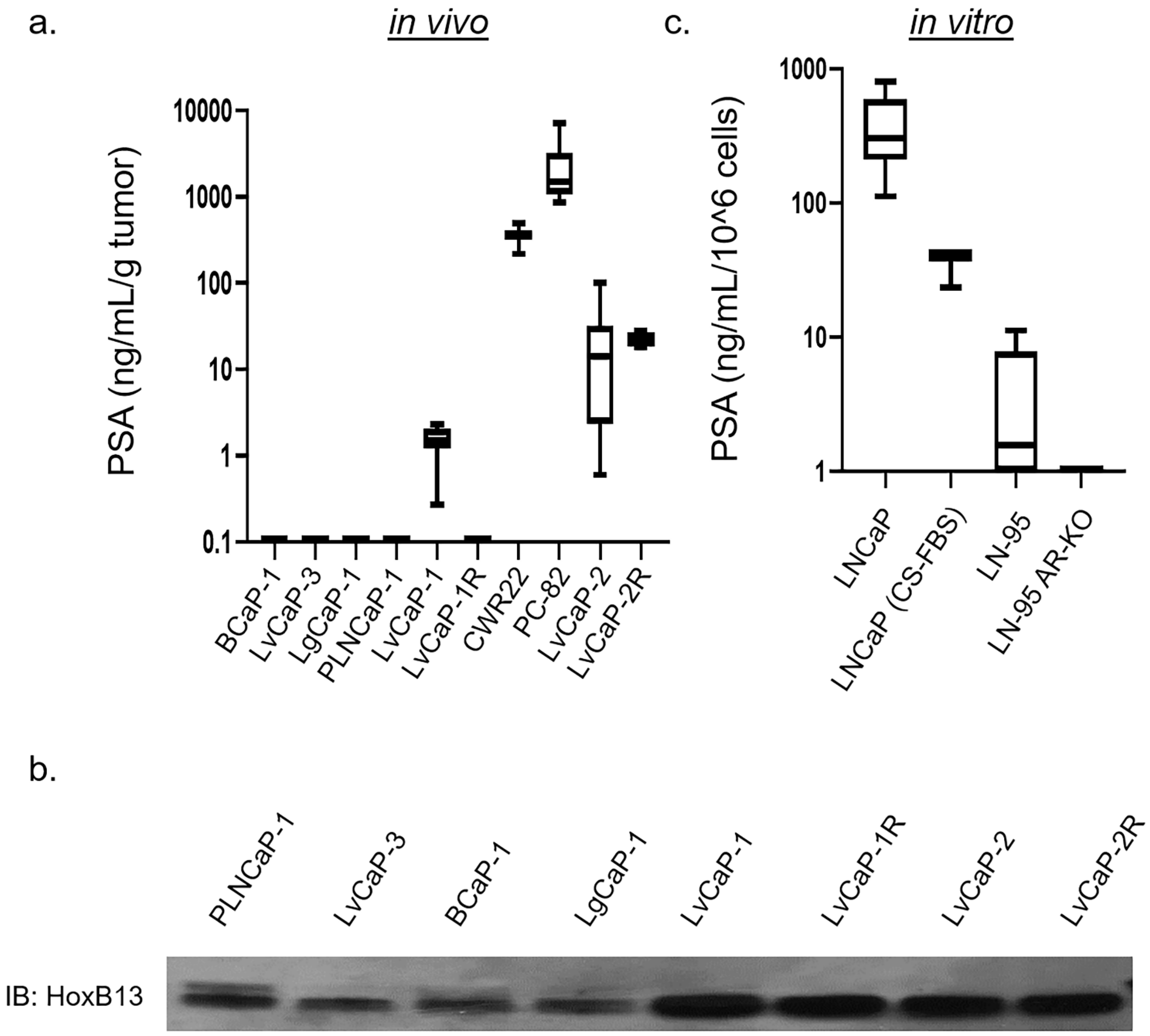

Supplemental Figure S2

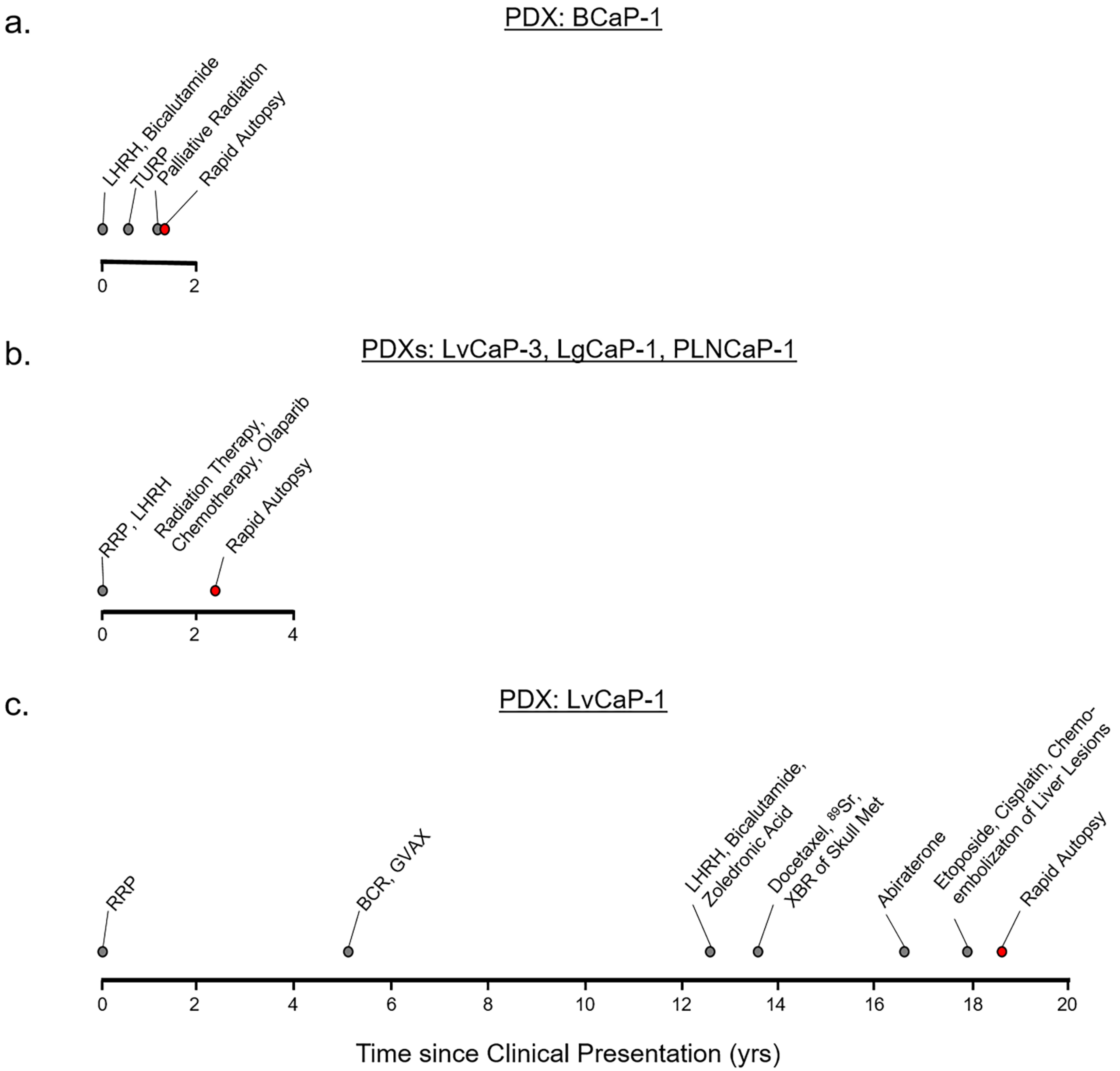

Supplemental Figure S3

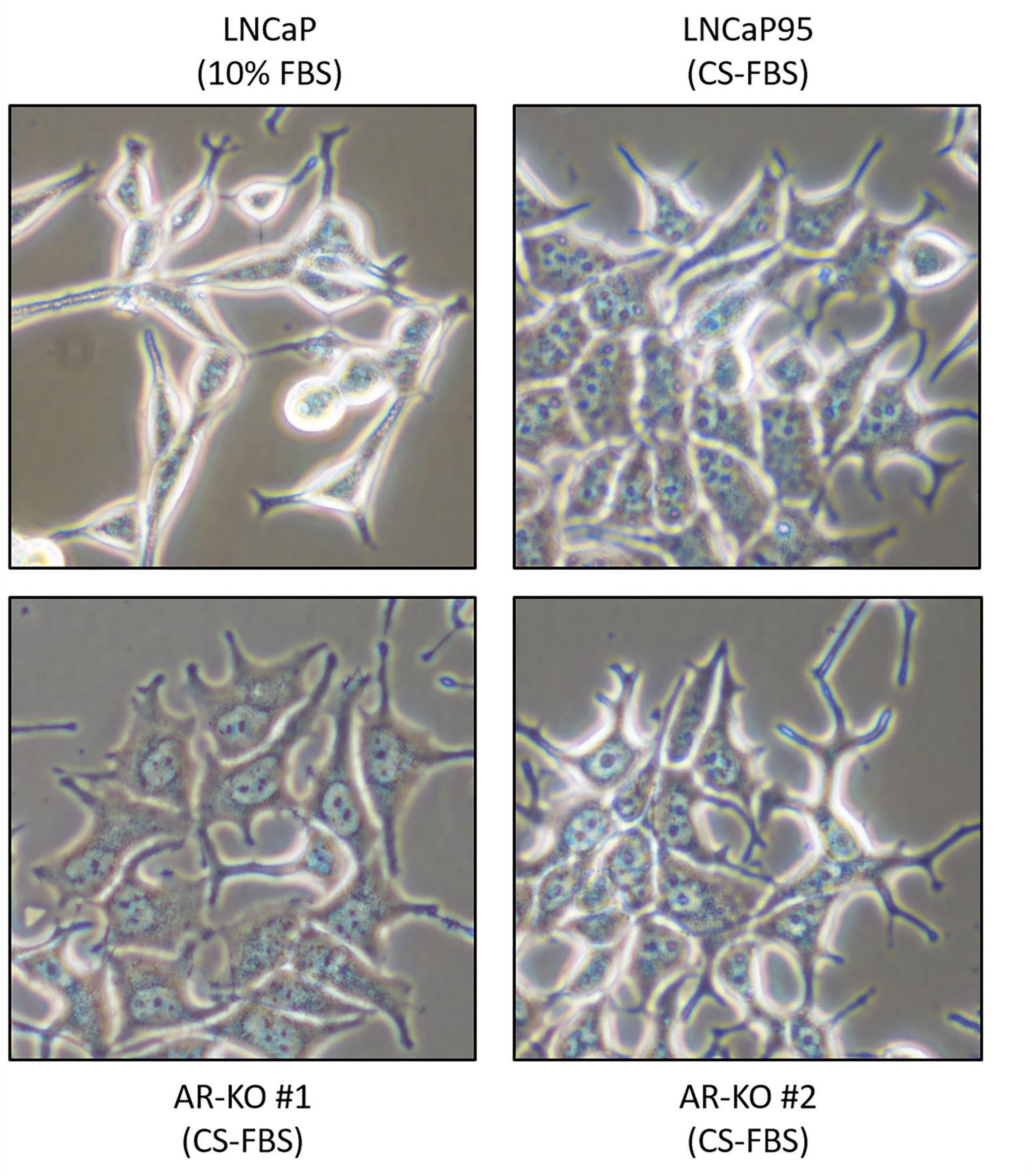

Supplemental Figure S4

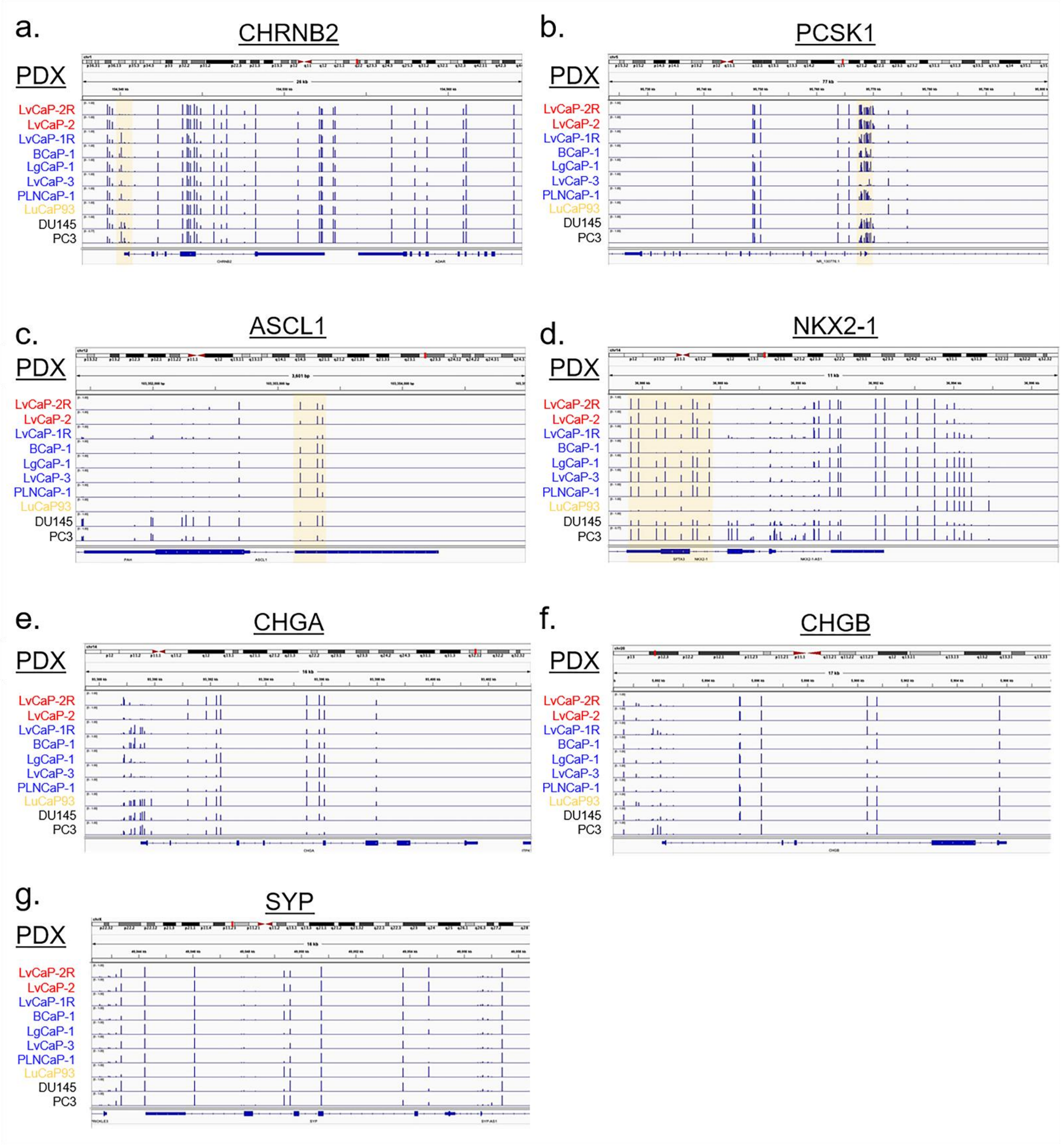

Supplemental Figure S5

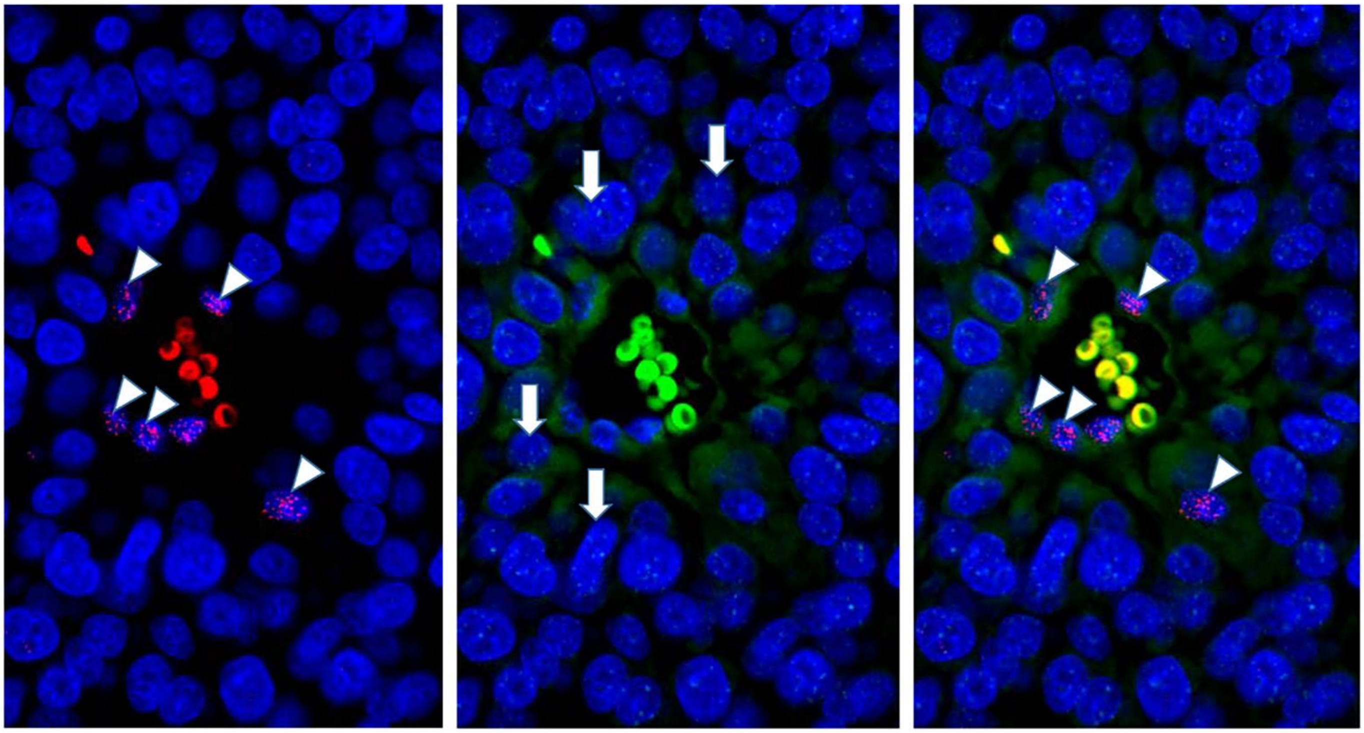

Supplement: Supplemental data [file jciinsight-6-146827-s140.pdf]
